# Preparing for the spread of patient-reported outcome (PRO) data collection from primary care to community pharmacy: a mixed-methods study

**DOI:** 10.1186/s43058-022-00277-3

**Published:** 2022-03-14

**Authors:** Omolola A. Adeoye-Olatunde, Geoffrey M. Curran, Heather A. Jaynes, Lisa A. Hillman, Nisaratana Sangasubana, Betty A. Chewning, David H. Kreling, Jon C. Schommer, Matthew M. Murawski, Susan M. Perkins, Margie E. Snyder

**Affiliations:** 1 Purdue University College of Pharmacy, 640 Eskenazi Avenue, Fifth Third Bank Building, Indianapolis, IN 46202 USA; 2 University of Arkansas for Medical Sciences College of Pharmacy, 4301 W. Markham St., #522-4, Little Rock, AR 72205-7199 USA; 3 University of Minnesota, College of Pharmacy, 308 Harvard Street, SE, Minneapolis, MN 55455 USA; 4 University of Wisconsin-Madison School of Pharmacy, 777 Highland Ave., Madison, WI 53704 USA; 5 Purdue University College of Pharmacy, 575 Stadium Mall Drive, Heine Pharmacy Building, West Lafayette, IN 47907 USA; 6 Indiana University School of Medicine, 410 W 10th Street, Indianapolis, IN 46202 USA

**Keywords:** Community pharmacy, Patient-reported outcomes, Health information technology

## Abstract

**Background:**

Medication non-adherence is a significant public health problem. Patient-reported outcomes (PROs) offer a rich data source to facilitate resolution of medication non-adherence. PatientToc™ is an electronic PRO data collection software originally implemented at primary care practices in California, United States (US). Currently, the use of standardized PRO data collection systems in US community pharmacies is limited. Thus, we are conducting a two-phase evaluation of the spread and scale of PatientToc™ to US Midwestern community pharmacies. This report focuses on the first phase of the evaluation. The objective of this phase was to prepare for implementation of PatientToc™ in community pharmacies by conducting a pre-implementation developmental formative evaluation to (1) identify potential barriers, facilitators, and actionable recommendations to PatientToc™ implementation and (2) create a draft implementation toolkit.

**Methods:**

Data collection consisted of demographics, observations, audio-recorded contextual inquiries, and semi-structured interviews with staff (e.g., primary care providers, pharmacists, pharmacy technicians) and patients during 1-day site visits to a purposive sample of (1) primary care practices currently using PatientToc™ and (2) community pharmacies in Indiana, Wisconsin, and Minnesota interested in the future use of PatientToc™. Post-visit site observation debriefs were also audio-recorded. Verbatim transcripts of all recordings were coded using deductive/inductive approaches and intra-/inter-site summaries were produced identifying potential barriers, facilitators, and actionable recommendations mapped to the Consolidated Framework for Implementation Research constructs. A stakeholder advisory panel engaged in an Evidence-Based Quality Improvement (EBQI) implementation process. This included “member checking” and prioritizing findings, and feedback on the adapted PatientToc™ application, implementation strategies, and accompanying toolkit for community pharmacy implementation.

**Results:**

Two primary care practices, nine pharmacies, and 89 individuals participated. Eight major themes (four barriers and four facilitators) and 14 recommendations were identified. Throughout the four EBQI sessions, the panel (1) confirmed findings; (2) designated high priority recommendations: (a) explain PatientToc™ and its benefits clearly and simply to patients, (b) ensure patients can complete questionnaires within 10 min, and (c) provide hands-on training/resources for pharmacy teams; and (3) provided feedback on the adapted PatientToc™ application and finalized toolkit items for initial community pharmacy implementation.

**Conclusions:**

Adoption of electronically captured PROs in community pharmacies is warranted. The implementation strategies systematically developed in this study can serve as a model for implementation of technology-driven health information patient care services, in the understudied context of community pharmacies.

**Supplementary Information:**

The online version contains supplementary material available at 10.1186/s43058-022-00277-3.

Contributions to the literature

- Patient-reported outcomes (PROs) offer a rich data source for pharmacists and prescribers to use to resolve medication non-adherence; however, no examples of widespread electronic collection and use of PRO data in community pharmacy settings exist.
- We conducted a pre-implementation developmental formative evaluation to inform implementation of an electronic PRO data collection software in community pharmacies.
- With the goal of optimizing identification and resolution of medication non-adherence, our findings provide actionable recommendations and an implementation toolkit for spreading an electronic PRO data collection software to community pharmacies.
- The resulting implementation toolkit could inform the implementation of other community pharmacy-based patient care services.

## Background

Medication non-adherence is a significant public health problem. Specifically, in older adults, medication non-adherence has been associated with all-cause hospitalization and mortality [1]. Community pharmacists are well-positioned to identify and intervene on medication non-adherence during medication counseling and other routine pharmacy patient care services [2]. Medication non-adherence may be influenced by negative medication-related outcomes that patients experience [3]. Patient-reported outcome (PRO) measures have become a standard in assessing patients’ behaviors towards treatment (e.g., medication adherence) [4]. Many PRO measures (e.g., Adherence Starts with Knowledge 20, General Medication Adherence Scale, Medication Adherence Reasons Scale) for medication non-adherence exist [5]. However, systematically collecting, documenting, tracking, and analyzing PROs can be problematic, particularly in the community pharmacy setting due to implementation challenges (e.g., meeting patient language needs, data privacy and security concerns, timely accessibility of data for pharmacist interventions) and limited use of standardized PRO data collection systems. Likewise, electronic patient-reported outcome data software addressing these challenges are more commonly used in other healthcare settings [4]; however, use in community pharmacy settings remains limited [6].

PatientToc™ is one type of PRO data collection software. It was initially developed by L.A. Net Community Health Resources Network investigators in California to meet the unique needs of diverse patients served by primary care practices [7]. The software facilitates PRO data collection using Android devices, with audio assist available in more than 200 languages [7]. A more detailed description and screenshots of PatientToc™ can be found in our study protocol paper [8]. The spread of this PRO data collection software from use in primary care to community pharmacies is being evaluated using a two-phase (pre-implementation followed by implementation) approach. The intended medication adherence-related PROs (i.e., responses to Brief Medication Questionnaire and Merck Medication Adherence Estimator) to be collected via PatientToc™ and plans for their use in phase two of the evaluation are provided in our study protocol paper [8]. The objectives of the study’s first phase, presented here, were to prepare for implementing PatientToc™ in community pharmacies by conducting a pre-implementation developmental formative evaluation to (1) identify potential barriers and facilitators, and actionable recommendations, to PatientToc™ implementation, and (2) create a draft implementation toolkit.

## Methods

### Design overview

The research team applied a convergent parallel, qualitatively driven mixed-methods [9] study design. This design equipped researchers to investigate expected barriers, facilitators, and actionable recommendations for PatientToc™ implementation in community pharmacies. Qualitative methods were the primary methods used for data collection and analysis. We intentionally provide extensive details of methods used to establish qualitative rigor (i.e., “trustworthiness”) in terms of credibility, transferability, dependability, and confirmability as defined with supporting accessible examples by Thomas and colleagues [10]. Quantitative methods were used to contextualize the study population and qualitative findings. Qualitative and quantitative data were analyzed concurrently and subsequently triangulated [11] via synthesis documents and group discussions (described in detail in the “Data synthesis” section). Reporting is in accordance with the Good Reporting of a Mixed Methods Study (GRAMMS) criteria (see Additional file 1) [12].

### Conceptual frameworks

The study design was guided by three conceptual frameworks:

1. Curran et al.’s approach to Evidence-Based Quality Improvement (EBQI) [13]
2. Consolidated Framework for Implementation Research (CFIR) [14]
3. Expert Recommendations for Implementing Change (ERIC) framework [15]

#### Application of framework #1

EBQI is a multilevel process to systematically incorporate scientific findings into healthcare settings [16, 17]. This process is motivated and facilitated by researcher and local stakeholder partnerships [16, 17]. Specific to this study, we adopted Curran’s two-step approach to EBQI:

1. Diagnosis of site-specific implementation needs, barriers, and facilitators (i.e., formative evaluation)
2. The use of multi-disciplinary teams of local staff, implementation experts, and clinical experts to interpret diagnostic data and develop/adapt site-specific interventions

EBQI also operates as an implementation strategy by enabling contextual adaptation of interventions and creating buy-in among stakeholders.

#### Application of framework #2

We applied the CFIR to guide qualitative data collection/analysis and data synthesis. The CFIR is a well-established determinant implementation framework that is comprehensive and well suited for complex, multilevel interventions. CFIR categorizes implementation constructs across five domains: intervention characteristics, outer setting, inner setting, characteristics of the individuals involved, and the process of implementation [14].

#### Application of framework #3

To further classify our actionable recommendations for PatientToc™ implementation, we applied Waltz et al.’s ERIC taxonomy of implementation strategies. The taxonomy suggests 73 discrete implementation strategies grouped into nine categories or “types” of strategies: use evaluative and iterative strategies, provide interactive assistance, adapt and tailor to context, develop stakeholder interrelationships, train and educate stakeholders, support clinicians, engage consumers, utilize financial strategies, and change infrastructure [15].

### Setting, site recruitment, and sampling

To address study objectives, the study setting consisted of Western primary care practices and Midwestern community pharmacies in the United States (US). The American Academy of Family Physicians defines primary care practice in the US as

the patient’s entry point into the health care system and as the continuing focal point for all needed health care services. Primary care practices are generally located in the community they serve, thereby facilitating access to health care while maintaining a wide variety of specialty and institutional consultative and referral relationships for specific care needs. The primary care practice structure often includes a team of physicians and other health professionals [18].

In addition to dispensing prescriptions (medications prescribed by an authorized provider), community pharmacists in the US are the most accessible healthcare professional to the public. Furthermore, US community pharmacies offer other pharmacy services including immunizations, disease state, and medication therapy management services. The community pharmacy structure typically includes licensed pharmacists and pharmacy technicians.

A purposive sample (*n* = 11) of study sites was recruited primarily through practice-based research networks (PBRN) across four states in the US: (1) (California) L.A. Net Community Health Resource Network (L.A. Net) [19], (2) Medication Safety Research Network of Indiana (Rx-SafeNet) [20], (3) Minnesota Pharmacy PBRN (MPPBRN) [21], and (4) select community pharmacies in Wisconsin. A description of each PBRN can be found in our protocol paper [8]. Typical recruitment practices (e.g., emails, phone calls) of each PBRN (mirrored for Wisconsin) were followed. To better understand how PatientToc™ has been implemented in primary care, we recruited two L.A. Net primary care practices in California, with varied approaches to PatientToc™ implementation. Three community pharmacies, consisting of a wide range of practice types and settings (e.g., urban vs. rural) from each of the remaining three states (nine total), were recruited to better understand potential barriers, facilitators, and recommendations for spreading PatientToc™ to community pharmacies.

### Qualitative data collection and analysis

A core team of investigators (OAAO, GMC, HAJ, MES) with qualitative research expertise participated in qualitative data collection activities across all study sites. Additional trained investigators (LAH, NS) participated in qualitative data collection at their respective state study sites. All researchers engaged in qualitative data collection participated in pre- and onsite study-specific training. Qualitative data collection consisted of audio-recorded semi-structured interviews, investigator observations of staff and patients at participating primary care and pharmacy sites, and contextual inquiries. Observations were conducted to provide insights into how PatientToc™ is currently used (primary care) and could be used (pharmacies). Contextual inquiries consisted of informal interviews with participants demonstrating and describing elements of their work duties (see Additional file 2). Lastly, to be reflexive of the research process, summary recorded debriefs following each site visit and resulting data were included as part of our formal data collection. We also debriefed (not part of data collection) after all site visits for each state were conducted. Our protocol paper provides additional details of each qualitative data collection method [8]. As mentioned previously, CFIR informed qualitative data collection. Specifically, semi-structured interview and observation guides were designed to explore participant experiences and probe for expected barriers, facilitators, and recommendations within the CFIR constructs. Contextual inquiry probes were designed to further explore specific routine tasks associated with PatientToc™ use by primary care stakeholders as well as pharmacy tasks expected to be influenced by future PatientToc™ use. By engaging with stakeholders as they completed routine tasks, the probes further elucidated expected barriers, facilitators, and recommendations.

Data collection forms were pilot tested and refined prior to use. Specifically, primary care data collection forms were pilot tested for content and process flow by three volunteers (one provider, one staff member, and one patient) familiar with PatientToc™. Pharmacy data collection forms were pilot tested for content and process flow by two volunteers (one community pharmacist and one community pharmacy patient) not familiar with PatientToc™. Minor modifications were made to all data collection forms for clarification purposes.

A purposive sample, targeting five clinicians/staff and five patients (18 years of age or older having at least one chronic condition for which they routinely take medication) from participating primary care and pharmacy practice sites, was invited to engage with researchers during 1-day site visits.

The qualitative analysis team included two trained student research assistants and an investigator (LAH). Audio-recorded semi-structured interviews, investigator observation summary debriefs, and contextual inquiries were transcribed verbatim (InfraWare Inc, Terre Haute, IN) and checked for accuracy. The qualitative analysis team coded transcripts using NVivo 12 Pro (QSR International). Both deductive (using CFIR) and inductive (emergent from the data) approaches were used in developing the codebook. The CFIR Codebook Template [22] was adapted to fit the context of our study and frame code definitions and coding inclusion/exclusion criteria. The final codebook is available in the supplemental files (see Additional file 3).

We detail our qualitative data coding steps below:

1. Deductive “level 1” broad codes consisted of the 41 CFIR constructs included in the CFIR Codebook Template [22].
  - Of note, several publications report 39 CFIR constructs [23]; however, we included all 41 listed in the CFIR Guide Codebook Template [22] in our codebook. Additional details regarding this approach to code development are provided in the supplemental files (see Additional file 3).
2. A total of 123 deductive sub-codes, termed “level 2” sub-codes, were included to further delineate each “level 1” broad code into potential barriers, facilitators, and actionable recommendations for PatientToc™ implementation in community pharmacies.
3. Subsequently, after the qualitative analysis team “level 2” coded transcripts, 234 inductive “level 3” codes were created to further delineate “level 2” codes in response to the actual data that were coded.

To illustrate this coding process, we will use the CFIR construct “Relative Advantage” as an example. Our “level 1” broad code was RelAdvantage. “Level 2” sub-codes were RelAdvantage_B, RelAdvantage_F, RelAdvantage_R, with B, F, and R indicating barrier, facilitator, and recommendation, respectively. Lastly “level 3” inductive sub-codes were created in response to the actual “level 2” coded data and included inductive codes such as Prefer paper_B, Easier_alternative_F, Increase_efficiency_R. Codes were modified, created, or collapsed as necessary [24].

Rotating pairs of analysts independently coded and reconciled the same transcripts for two study sites, until all transcripts for these sites were reconciled. Subsequently, analysts independently coded an approximately equal number of different transcripts for the remaining study sites. No double coding (i.e., using more than one code to code the same pieces of text) was permitted. Three other investigators (OAAO, HAJ, MES) with expertise in qualitative research reviewed approximately one-third of all coded transcripts and met with the qualitative analysis team regularly to provide feedback and ensure the codebook was applied consistently.

### Quantitative data collection and analysis

Quantitative data collection consisted of the study site and participant demographics (see Additional file 2). Study site demographics, e.g., type of practice, number and type of staff members, prescription volume, were self-reported from the primary site contact and collected by telephone in advance of the 1-day site visits. Participant demographics, e.g., age, role, race, years employed at practice site, were self-reported and collected at the end of each participant’s interview. All quantitative data were collected and managed using Research Electronic Data Capture (REDCap™) [25] electronic tools hosted at the Indiana Clinical and Translational Sciences Institute. REDCap™ is a secure, Web-based application designed to support data capture for research studies, providing (1) an intuitive interface for validated data entry; (2) audit trails for tracking data manipulation and export procedures; (3) automated export procedures for seamless data downloads to common statistical packages; and (4) procedures for importing data from external sources [25]. Site and participant demographics were summarized by computing descriptive statistics (counts and percentages, means, and standard deviations) at the site level, and then per site descriptive statistics were used to create a mean across all sites using SPSS v. 25 (IBM Corp).

### Data synthesis

*Intra*-site summary documents were created to summarize qualitative and quantitative findings for each participating primary care practice and pharmacy. Mixing and triangulation [11] consisted of the qualitative analysis team noting convergence or salient differences in qualitative findings by qualitative method used (e.g., if investigator observation summary debrief data confirmed or differed from interview data*)* and select quantitative variables, site, and participant type. Informed by *intra*-site summary documents, an *inter*-site summary document was created to facilitate investigator synthesis and identification of final themes during planned research team discussions. During the course of 2 days (13 h total), research team members met virtually (due to COVID-19 travel restrictions) via videoconferencing software to review *intra*- and *inter*-site synthesis documents and identify themes by CFIR construct. The principal investigator (MES) compiled and summarized notes from the research team discussions to identify overarching major themes, categorized as barriers or facilitators and recommendations mapped to applicable CFIR constructs. Research team members who participated in the 2-day group discussions had opportunities to review the major themes/recommendations and accuracy of mapping to CFIR constructs. Minor modifications (e.g., re-mapping of a few CFIR constructs) were made through the team review process. Mixing and triangulation [11] consisted of noting convergence or salient differences in final major themes by type of practice and participant role.

### EBQI process and draft implementation toolkit

Resulting major themes informed the EBQI process for this study. This process consisted of assembling a multi-stakeholder advisory panel. In addition to a subset of study team members, the target number of Advisory Panel members was nine stakeholders—a pharmacist, a technician, and a patient from each state (i.e., one participant of each stakeholder type representing each pharmacy study site). In selecting stakeholders to invite, we attempted to balance demographics including gender, race, number of medications taken, and frequency of pharmacy visits (patients), as well as expected contributions/willingness to share ideas (based on interview responses), and perceived engagement with the site/project (for pharmacists/staff, based on site visits). For this phase of the study, we held a total of four, 120-min virtual EBQI sessions via videoconferencing software. To gather the unique perspectives of pharmacy staff and patients, “breakout” groups were held during the sessions to discuss priority questions relevant to each stakeholder group. Their insights were then shared with the whole group. The first three sessions consisted of member checking and prioritizing findings. Panelists’ initial recommendations from these sessions informed (1) mockup of the adapted PatientToc™ application and (2) draft toolkit resources for initial spread of PatientToc™ in community pharmacies. These items were reviewed during the fourth EBQI session and panelist recommendations for PatientToc™ adaptations and implementation were finalized.

## Results

Keeping the study objective at the forefront and for confidentiality purposes (given small sample sizes), we first present a brief description of the current PatientToc™ implementation context at participating primary care practices (*n*=2) followed by a summary of quantitative, qualitative, and synthesis (barriers and facilitators) findings from participating pharmacies (*n*=9). Finally, we provide the actionable recommendations and the implementation toolkit items drafted in response and created during the EBQI process.

### PatientToc™ implementation at participating primary care practices

Across the two primary care practices, a total of two providers (e.g., doctor, nurse practitioner), six support staff (e.g., registered nurses, medical assistants), and two patients participated. Both primary care practices serve a diverse patient population including Hispanic/Latino/Latinx/Spanish, Black/African-Americans, socioeconomically disadvantaged, medically underserved, Medicaid or dual Medicaid/Medicare beneficiaries, adult and pediatric patients, and pregnant women receiving pre-natal care. PatientToc™ implementation varied across the participating practices. One practice used it to collect survey data from Consumer Assessment of Healthcare Providers and Systems (CAHPS®), a federal program that administers surveys to capture and evaluate patient experiences, and use of the tablets has become well integrated into the facility’s workflow. Currently, the PatientToc™ tablets are mounted on rolling carts and patients’ complete questionnaires in private after being “roomed” and waiting for their provider. Many of the primary care providers and support staff are familiar with the tablet. The practice also implemented competitive incentives associated with the number of CAHPS® completed on PatientToc™.

The other practice currently stations PatientToc™ tablets in the waiting area at dedicated tables but signs indicate that tablets should not be used by patients unless instructed by staff. We did not observe tablet use by staff or patients during our visit. At this site, PatientToc™ is being used to capture “Staying Healthy Assessments,” which are part of the required California Medicaid (Medi-Cal) Initial Health Assessment. Questions focus on topics such as pain, depression, and fall risk. One L.A. Net staff member is embedded in the practice to assist with PatientToc™ implementation. While some of the primary care providers were familiar with PatientToc™, support staff appeared to have minimal familiarity.

### Quantitative, qualitative, and synthesis findings from participating pharmacies

Table 1 summarizes pharmacy and participant characteristics at the site level and by participant type. A total of nine pharmacies participated in this pre-implementation phase of the study. The sites consisted of independent (*n*=6, 67%) and health system (*n*=3, 33%) pharmacies. In the US, independent pharmacies are privately held retail pharmacies not owned or operated by a publicly traded company and have no affiliation with any chain of pharmacies. For the purposes of this study, we utilized the National Council for Prescription Drug Programs (NCPDP) definition of independent pharmacy, namely one to three pharmacy locations under common ownership [26]. Likewise, a health system pharmacy is a retail pharmacy that is affiliated with a health system, which is defined as an organization that includes at least one group of providers who provide primary and/or specialty care that is integrated with each other and the hospital through joint management or common ownership. Across all sites, the mean (standard deviation (SD)) weekly prescription volume was 1266 (605) and had a mean (SD) of 3 (1) full-time equivalent pharmacists and 6 (5) full-time equivalent staff. All pharmacies offered 90-day prescription fills as a service to facilitate patients’ adherence to prescription medications. Across the nine community pharmacies, a total of 22 pharmacists and 28 pharmacy staff (e.g., pharmacy technicians, service clerks) participated and reported the mean (SD) weekly percent of hours spent working with patients as 80% (15) and 84% (19), respectively. A total of 34 pharmacy patients participated in the study. Pharmacy patients participants primarily identified as non-Hispanic White (mean (SD) 88% (22)), half (50% (30)) identified as male, 31% (37) visited their pharmacy at least once a week, and regularly used eight medications on average.

**Table 1:** Characteristics of participating community pharmacies (*n*=9), pharmacists (*n*=22), pharmacy staff (*n*=28), and patients (*n*=34)^a^

Site-level characteristics (***n***=9)	
Variable	Result
Type of pharmacy, *n* (%)	
Independent (< 4 locations)	6 (67%)
Health-system outpatient	3 (13%)
Number of pharmacist FTEs, mean (SD)	3 (1)
Number of staff FTEs, mean (SD)	6 (5)
Weekly prescription volume, mean (SD)	1266 (605)
Types of medication adherence services offered, *n* (%)	
Delivery	8 (89%)
Auto-refill	5 (56%)
Compliance packaging	7 (78%)
Medication synchronization	6 (67%)
Participation in EQuiPP	8 (89%)
Mobile app	4 (44%)
90-day fills	9 (100%)
Other^b^	7 (78%)
Patient care services offered, *n* (%)	
Medication therapy management	8 (89%)
Diabetes management	3 (33%)
Other^c^	9 (100%)
Workflow features, *n* (%)	
Drive-through window	2 (22%)
**Subject-level characteristics (summarized at site level)**	
***Number of pharmacist participants***	22
Per site, number of pharmacist participants in the study, mean (SD)	2 (1)
Per site, pharmacist age, mean (SD)	38 (10)
Per site, pharmacist percent male, mean (SD)	39 (24)
Per site, pharmacist percent not Hispanic, mean (SD)	96 (11)
Per site, pharmacist percent white, mean (SD)	94 (17)
Per site, pharmacist percent PharmD, mean (SD)	78 (36)
Per site, pharmacist, percent with no residency training, mean (SD)	83 (25)
Per site, pharmacist, years employed at this pharmacy (any position), mean (SD)	6 (4)
Per site, percent of pharmacist participants, current position at pharmacy, mean (SD)	
Owner, mean (SD)	28 (36)
Manager, mean (SD)	15 (23)
Staff pharmacist, mean (SD)	34 (29)
Per site, pharmacist, percent of working hours working with patients within a week, mean (SD)	80 (15)
***Number of pharmacy staff participants***	**28**
Per site, number of pharmacy staff participants in the study, mean (SD)	3 (1)
Per site, pharmacy staff, age, mean (SD)	41 (8)
Per site, pharmacy staff percent male, mean (SD)	9 (19)
Per site, pharmacy staff, percent not Hispanic, mean (SD)	97 (8)
Per site, pharmacy staff, percent white, mean (SD)	89 (19)
Per site, pharmacy staff, percent pharmacy technicians, mean (SD)	89 (14)
Per site, pharmacy staff, percent highest degree completed, mean (SD)	
High school (GED)	33 (41)
Per site, pharmacy staff, years employed at this pharmacy (any position), mean (SD)	6 (6)
Per site, pharmacy staff, years employed as a pharmacy staff member at this pharmacy, mean (SD)	7 (7)
Per site, pharmacy staff, percent hours working with patients per week, mean (SD)	84 (19)
***Number of pharmacy patient participants***	**34**
Per site, number of patient participants in the study, mean (SD)	4 (1)
Per site, patient age, mean (SD)	63 (8)
Per site, patient percent male, mean (SD)	50 (30)
Per site, patient percent not Hispanic, mean (SD)	100 (0)
Per site, patient percent white, mean (SD)	88 (22)
Per site, patient percent, frequency of pharmacy visit, mean (SD)	
At least once a week	31 (37)
Less than every week but more than once a month	11 (21)
About once a month	26 (22)
About once every 3 months	16 (21)
Per site, patient number chronic conditions that require a routine prescription, mean (SD)	3 (1)
Per site, patient number of prescription medications regularly used, mean (SD)	6 (2)
Per site, patient number of over the counter medications regularly used, mean (SD)	1 (1)
Per site, patient number of supplements regularly used, mean (SD)	2 (1)
Per site, patient number of total medications regularly used, mean (SD)	8 (2)

*Abbreviations*: *SD* Standard deviation, *FTE* Full-time equivalent, *EQuiPP* Electronic Quality Improvement Platform of Plans and Pharmacies, *MTM* Medication therapy management

^a^Site demographic data were self-reported from the research team’s primary contact person at the site; subject-level demographic data were self-reported by the subject. No attempts were made to verify data from other sources (e.g., the EMR/dispensing system for patient/prescription volume or number of reported prescriptions taken by patients)

^b^Responses to “other” included website available for refills, refill reminder calls, targeted disease state adherence checks/ calls, mail prescriptions, texting service for refill reminders, and “Flip the Pharmacy” initiative

^c^Responses to “other” included vaccines, blood pressure monitoring cholesterol monitoring, point of care testing, medical equipment fitting/ supply, travel health consulting, weight management, home care services, and naloxone consulting

Table 2 lists major themes (and associated theme description, representative quotations, and CFIR constructs) categorized as expected barriers and facilitators for PatientToc™ implementation in community pharmacies. We identified a total of 8 major themes: four barriers and four facilitators, for PatientToc™ implementation in community pharmacies. Convergence of qualitative results was evident across all qualitative data collection methods (semi-structured interviews and contextual inquiries with participant and investigator observation debriefs); thus, all illustrative quotations are from qualitative semi-structured interview data and included nuances by select quantitative variables including site type [(1) independent pharmacies, (2) health system pharmacies, (3) both pharmacy types, (4) primary care, (5) all primary care and pharmacy types] and participant type [(1) pharmacy staff (including staff and pharmacists), (2) pharmacy patients, (3) primary care staff (including providers and staff), (4) primary care patients, (5) primary care and pharmacy staff, (6) primary care and pharmacy staff and patients].

**Table 2:** Major themes, descriptions, and participant quotations categorized as potential barriers and facilitators for PatientToc™ Implementation^a^

Major themes pertaining to community pharmacy implementation	Theme descriptions and representative quotations^**b**^ by applicable site^**c**^ and participant^**d**^ type	Related CFIR constructs
**Potential barriers**		
1. Lack of *existing integrations among technology vendors* and/or concerns about the feasibility/effectiveness of future integrations of existing pharmacy technology with PatientToc™.	Some pharmacy staff expressed concerns about integrating PT with dispensing systems (both pharmacy types) or electronic health records (health system pharmacies).	Adaptability, Cosmopolitanism, Networks & Communications, Compatibility, Available Resources, External Change Agents
	“Firewalls are a big concern with integrating [PT]... with multiple software … dispensing software, outpatient clinic software and inpatient software.” – Health system pharmacy (staff)	
2. Some *sub-groups of patients* (e.g., older adults, those with arthritis, those who do not physically come in to the pharmacy, those who prefer paper over technology) might be challenged to use PatientToc™.	Some pharmacy patients expressed concerns over technological complexity, managing PT, and seeing how it meets a need for them while pharmacy staff described concerns due to patient age and co-morbidities and needing to walk patients through it (both pharmacy types).	Relative Advantage, Complexity, Design Quality & Packaging, Patient Needs & Resources, Structural Characteristics, Culture, Compatibility, Engaging, Intervention Participants
	“There would be some people who would be... you know, we have a pretty elderly clientele. They would look at something like this and say, no, thank you.” – Independent pharmacy (staff)	
3. PatientToc™ could be difficult to incorporate into *pharmacy workflow* due to space (e.g., small waiting areas, shared space) or staffing (e.g., time required, possible need for additional staff, competing demands) constraints.	Some pharmacy staff expressed concern with available space for pharmacy patients to use PT while many pharmacy staff noted a potential need for additional staff time due to constraints and competing demands (both pharmacy types).	Structural Characteristics, Patient Needs & Resources, Implementation Climate, Compatibility, Available Resources
	“Some of your elderly population come in with their cell phones and they are using them for things, or they are calling in or using the internet to call in the refills, but then there are other ones that absolutely won't use our automated system and want to talk to somebody all the time, so I think those people would probably need more help and from a workflow standpoint, I don't know how much extra time we would have to walk them all the way through... so that would be a concern.” – Health system pharmacy (staff)	
4. *Data security* concerns (e.g., privacy of information provided in PatientToc™ by patients, mistrust of technology, uncertainty regarding where the information is sent) could limit uptake of PatientToc™ by pharmacy patients.	There are some concerns that patients might resist or not trust technology due to concerns with privacy and/or a general mistrust in technology (all primary care practices and pharmacy types) (primary care and pharmacy staff and patients).	Adaptability, Complexity, Patient Needs & Resources, Intervention Participants
	“I like to talk face-to-face. I don’t trust these things… Everybody can get your information on them.” – Independent pharmacy (patient)	
	“There is always some reluctance to everything, because everyone... now they have everyone afraid about data... so everyone is like, what are you going to do with my data? What are you going to do with my data? You know, so that is going to be your biggest challenge... everyone is like, oh you are going to get rich off my data, you know, I mean... just the fact that the media has sort of undermined the credibility… everybody is a suspect now.” – Primary Care (staff)	
**Potential facilitators**		
1. Pharmacy teams are generally willing to try new things, like PatientToc™, if it will help advance their *number one goal of improving patient care*.	There is general optimism for PT and expected buy-in from pharmacy staff in trying new things that will benefit and be perceived to be of value to patient care (both pharmacy types) (pharmacy staff).	Evidence Strength & Quality, Culture, Implementation Climate, Organizational Incentives & Rewards, Learning Climate, Readiness for Implementation, Knowledge & Beliefs About the Intervention, Individual Stage of Change, Individual Identification with Organization
	“I think most of us are pretty open to trying new things. Um... I think on a more global scale, it sort of matches with what independent pharmacy is trying to do, which is to provide a better patient experience, you know, and that is sort of an all-encompassing thing from [the] start of getting the [medication] orders, getting orders changed when they need to be, to actually providing real caring consults for people.” – Independent pharmacy (staff)	
2. Pharmacy *leadership is respected and generally strong communication* across team members is present, which would support PatientToc™ implementation.	There is a strong sense that leadership is supportive and respected and motivation to do well was noted with many sites. Teamwork is reflected in regularly scheduled meeting times (all primary care practices and pharmacy types) (primary care and pharmacy staff).	Networks & Communications, Leadership Engagement, Individual Identification with Organization, Opinion Leaders
	In specific, pharmacy staff generally view technicians as equals and have a good understanding of roles (both pharmacy types), and there is evidence of strong pharmacy-patient relationships in which patients feel motivated to help the pharmacy (both pharmacy types) (pharmacy patients).	
	“I think that helps because I am here on a daily basis and not only do I help in, you know, improving numbers, but I also help in other areas of the workflow… and then just building the rapport with the team, knowing that, you know, I hear them on a daily basis and they can come to me with anything, open door policy, and just... yeah, I have a good rapport with my team.” – Primary Care (staff)	
	“Well, we all do our huddles. The pharmacist is very encouraging and very helpful, and so, like after the huddle, everyone just feels pumped, and so, ... it feels... because we all get together and we talk about how we could all improve within the pharmacy...” – Health system pharmacy (staff)	
3. *Measures of importance to pharmacy teams* (e.g., Medicare Star ratings, CPESN USA metrics, patient satisfaction, medication adherence) *align with those expected to be impacted by PatientToc™* and measured by the research team.	Most pharmacy staff expressed agreement that there are multiple sources of alignment with quality performance metrics of importance to the pharmacy and PT that also align with research plans and objectives (both pharmacy types) (pharmacy staff).	Evidence Strength & Quality, Costs, External Policies & Incentives, Relative Priority, Goals & Feedback, Reflecting & Evaluating
	“…system-wide it would help out a lot. I mean, at our site, we do adherence, but I don't know if we do as much as all the other sites just because of our patient population, so I guess it would align with our [company] pharmacy overseeing umbrella goals very well.” – Health system pharmacy (staff)	
	“So, I think one big thing we talk about is like Star measures and the quality measures. So, …if this could help us with that, um, that would be a big driver to help implement it.” – Health system pharmacy (staff)	
4. Most stakeholders (pharmacists, pharmacy staff, and patients) felt PatientToc™ was *easy to use, felt training requirements would be minimal, and offered limited suggestions* for improvement.	Most pharmacy participants felt PT flowed and worked well and generally felt confident in their ability to use it with any improvements for suggestion likely feasible (both pharmacy types) (pharmacy staff and patients).	Complexity, Design Quality & Packaging, Access to Knowledge & Information, Self-Efficacy
	“I think, honestly, you’ve overcome all the barriers as far as navigating the tablet. I don’t think you could make it any more simple.” – Independent pharmacy (staff)	
	“…it’s just the print was just right, the lettering was easy to read, the font was okay and the colors were right, the display was easy at a glance you could understand it, so...Even if you are not computer literate, it might take you a minute, but you could figure it out eventually.” – Independent pharmacy (patient)	

*Abbreviations*: *CFIR* Consolidated Framework for Implementation Research, *CPESN* Community Pharmacy Enhanced Services Network, *PT* PatientToc™

^a^Sample includes all 11 participating pharmacy (*n*=9) and primary care practice (*n*=2) sites

^b^Convergence of qualitative results was evident across all qualitative data collection methods (semi-structured interviews and contextual inquiries with participants and investigator observation debriefs); thus, theme descriptions and supporting quotations were informed by semi-structured interview data only

^c^Site type was categorized as follows: (1) independent pharmacies, (2) health system pharmacies, (3) both pharmacy types, (4) primary care, and (5) all primary care and pharmacy types

^d^Participant type was categorized as follows: (1) pharmacy staff (including staff and pharmacists), (2) pharmacy patients, (3) primary care staff (including providers and staff), (4) primary care patients, (5) primary care and pharmacy staff, and (6) primary care and pharmacy staff and patients

Of the four barrier-related themes, two included potential PatientToc™ integration challenges with existing pharmacy technology and within workflow. The other two barrier-related themes included potential challenges with uptake by certain patients due to concerns with data security and preferences (e.g., those who prefer providing information on paper compared to using technology). Of the four facilitator-related themes, three included pharmacy staff and leadership factors including pharmacy staff members’ willingness to try PatientToc™ if it can potentially improve patient care, respected leadership and strong communication across pharmacy team members (supporting PatientToc™ implementation), and pharmacy-related measures aligning with those expected to be impacted by PatientToc™. The last facilitator-related theme was the perceived ease of PatientToc™ use by both pharmacy staff and patients.

### Recommendations and development of implementation toolkit items

A total of 14 actionable recommendations were identified (Table 3). The multi-stakeholder advisory panel was comprised of community pharmacists (*n*=3), pharmacy staff (*n*=2), and patient (*n*=3) participants representing seven (77.8%) of the participating pharmacy sites. Throughout three EBQI sessions, the multi-stakeholder panel confirmed the thematic findings and indicated the highest priority recommendations: (a) provide clear and simple instructions to patients that emphasize the expected benefits of PatientToc™, (b) ensure patients can complete questionnaires within 10 min, and (c) provide hands-on training/resources for pharmacy teams. Toolkit items were drafted in response. In the fourth EBQI session, panelists reviewed a mock-up of the PatientToc™ adapted for use in community pharmacies. Toolkit items for initial community pharmacy implementation were finalized. Four recommendations pertaining to intervention adaptations will be addressed in the final PatientToc™ build during phase two. Table 3 lists all of the 14 recommendations made by type of participant (pharmacy staff, patient, both) and associated ERIC implementation strategies [15] and describes specific strategy/toolkit resources.

**Table 3:** Summary of recommendations, implementation strategies^a^, and initial PatientToc™ implementation toolkit developed through the EBQI process^b^

Recommendations made by type of participant (pharmacy staff, patient, both)	Implementation strategy^**a**^	Specific toolkit item/strategy	Description
1. Ensure *all pharmacy team members* (pharmacists, technicians/other support staff, management) are *engaged/bought-in* to implementation of PatientToc™. (Staff)	*Develop stakeholder interrelationships: conduct local consensus discussions*	- PatientToc™ Mission Statement Template - Kickoff Agenda Template - Have check-in meetings - Audit and Feedback Report Template	*PatientToc™ Mission Statement* document is intended to create buy-in. The template is guided by the following three questions and includes example responses. The three questions are as follows: 1. Why are we using PatientToc™? 2. How will we use it in our pharmacy? 3. What goals are we trying to meet with PatientToc™? Example statement: “At [insert pharmacy name] pharmacy, our mission for using PatientToc™ is to [insert answer(s) to question 1]. We will accomplish this mission by using PatientToc™ to [insert answer(s) to question 2]. In using PatientToc™, our goals are to [insert answer(s) to question 3]. *Kickoff Agenda* document provides an overview of training and site preparation activities/resource tools. Develop and deploy routine *Check-in* Meetings to be held weekly via alternating phone/Web-based calls and in-person site visits. Provide *Audit and Feedback* report of key measures and problem-solving activities. Examples of measures in the report include number of patients using PatientToc™, where PatientToc™ is being used in the workflow (e.g., pharmacy waiting area, during appointment, outside of pharmacy with delivery driver)
2. Have clear PatientToc™ implementation *goals, measure outcomes* (e.g., adherence, patient satisfaction, pharmacist interventions made, ROI), and *provide feedback on outcomes and progress toward goals*. (Staff)	*Use evaluative and iterative strategies: audit and provide feedback*	- PatientToc™ Mission Statement Template - Have check-in meetings - Audit and Feedback Report Template	*For the most part, implementation goals and evaluation outcomes are set by the study team but align with measurement outcomes desired by staff.*
			Goals will be included in each pharmacy-specific *PatientToc™ Mission Statement* document.
			*Audit and Feedback* Document and routine *Check-In Meetings* to share data and problem-solve.
3. Explore the *use of incentives* for pharmacy team members (e.g., bonuses, food, praise) and/or patients (e.g., coupons, food, gift cards) to support PatientToc™ implementation. (Both)	*Utilize financial strategies: alter incentive/allowance structures*	Not applicable	*Incentives will not be provided by the study team as part of the implementation toolkit; individual pharmacies may choose to use incentives for their staff.*
4. Provide hands-on training and resources for pharmacy teams, possibly for continuing education credit, to support PatientToc implementation. (Staff)^c^	*Train and educate stakeholders:* *• Conduct ongoing training* *• Provide ongoing consultation* *• Develop educational materials* *• Make training dynamic* *• Distribute educational materials* *• Conduct educational outreach visits* *• Work with educational institutions*	- PatientToc™ Training Modules - Social Determinants of Health (SDOH) Continuing Education (CE) modules	There will be both required and optional *PatientToc™ Training Modules* on topics including initial tablet set-up and troubleshooting, overview of PROs being captured, use of delivery drivers, reports, and documentation.
			Per request by the multi-stakeholder advisory panel, *SDOH CE modules were created* and will be accredited through collaboration with a college of pharmacy continuing education office, as optional training for both pharmacists and pharmacy technicians. This will provide information on how to identify and address SDOH barriers based on PatientToc™ responses from patients.
5. Work with pharmacy teams and vendors to ensure PatientToc™ is *well integrated* with the pharmacies’ dispensing systems. (Staff)	*Adapt and tailor to context: promote adaptability*	Not applicable	Not applicable, part of intervention development/finalized build of adapted PatientToc™ application for use in community pharmacies.
	*Provide interactive assistance: provide local technical assistance*		
6. Ensure a PatientToc™ 24/7 *help line* is available to pharmacy teams. (Staff)	*Provide interactive assistance: centralize technical assistance*	Not applicable	-Availability/way of reaching PatientToc™ will be provided as part of training; however, a 24/7 help line is not currently available.
7. Consider adapting PatientToc™ for *more languages*. (Both)	*Adapt and tailor to context: promote adaptability*	Not applicable	Not applicable, part of intervention development/finalized build of adapted PatientToc™ application for use in community pharmacies (when needed).
8. Provide *clear and simple messaging to patients that emphasize the expected benefits* of PatientToc™ to patients. These messages should be provided (1) verbally by pharmacy staff, (2) written as part of introductory instructions in the PatientToc™ application, and/or (3) written as part of general pharmacy marketing materials (e.g., websites, waiting room televisions). (Both)^c^	*Engage consumers: prepare patients/consumers to be active participants*	- Patient-facing print (large and small posters, pamphlets, bag stuffers) and digital media (social media posts) materials - Provide scripted language for pharmacies’ use	Provide *patient-facing print and digital media materials*, as well as *scripted language* for pharmacies to use on websites, signs, TVs, as introduction scripts for staff, etc.
			Clarity across messaging (brochures, etc.) that PatientToc™ is private and expected to “help the pharmacy to better help the patient.”
9. Implement PatientToc™ first with *specific patient sub-groups* (e.g., complex patients). (Both)	*Engage consumers: prepare patients/consumers to be active participants*	Not applicable	Not applicable, for study evaluation purposes, the study cohort has already been determined; pharmacies will choose scope/where in their workflow they will implement PatientToc™.
10. *Enable access to PatientToc™ in various ways* (e.g., tablet in adjacent primary care practice site, delivery drivers bring a tablet, patient uses application on their own device) based on pharmacy workflow/patient needs. (Both)	*Change infrastructure:* *• Change physical structure and equipment* *• Change service sites*	- Sample workflows - Workflow cheat sheets	The study team has decided that pharmacies will get to choose where/how in their workflow they use PatientToc™.
			*Sample Workflows* for various use cases chosen and staffing patterns at the pharmacy.
			*Cheat Sheets* for each staff member per workflow option describing roles, activities, sample language to use with patient, etc.
11. Ensure patients can complete PatientToc™ questionnaires in 2–10 min (e.g., pre-populate information when possible, reduce the need for typing). (Both)^c^	*Engage consumers: prepare patients/consumers to be active participants*	Not applicable	Not applicable, part of intervention development/finalized build of adapted PatientToc™ application for use in community pharmacies.
12. Use PatientToc™ to *optimize patient prescription wait times as well as other appointment-based services* (e.g., MTM, medication synchronization program). (Both)	*Engage consumers: prepare patients/consumers to be active participants*	- Sample workflows - Workflow cheat sheets	The study team has decided that pharmacies will get to choose where/how in their workflow they use PatientToc™.
			*Sample Workflows* for various use cases chosen and staffing patterns at the pharmacy.
			*Cheat Sheets* for each staff member per workflow option describing roles, activities, sample language to use with patient, etc.
13. Use PatientToc™ to *update patient demographics (e.g., contact information, allergies, etc.)* and medication lists. (Both)	*Support clinicians: support relay of clinical data to providers*	Not applicable	Not applicable, part of intervention development/finalized build of adapted PatientToc™ application for use in community pharmacies.
14. Consider including *patient education and/or information/referrals* to pharmacy services on PatientToc™. (Both)	*Adapt and tailor to context: promote adaptability*	- Referrals Cheat Sheet	The *Referrals Cheat Sheet* provides a modifiable template of resources for pharmacy teams to consider when reviewing PatientToc™ results to identify and address medication non-adherence.

*Abbreviations*: *CE* Continuing Education, *EBQI* Evidence-Based Quality Improvement, *MTM* Medication therapy management, *SDOH* Social Determinants of Health

^a^Implementation strategies are per Waltz TJ, Powell BJ, Matthieu MM, Damschroder LJ, Chinman MJ, Smith JL, et al. Use of concept mapping to characterize relationships among implementation strategies and assess their feasibility and importance: results from the Expert Recommendations for Implementing Change (ERIC) study. Implement Sci. 2015;10(1):109

^b^EBQI process was per Curran GM, Mukherjee S, Allee E, Owen RR. A process for developing an implementation intervention: QUERI Series. Implement Sci. 2008;3(1)

^c^Multi-stakeholder advisory panel (pharmacy staff and patients) designated recommendation as one of the top 3 highest priority recommendations

Across the 14 actionable recommendations, there was alignment with one or more of the 9 ERIC implementation strategy categories. Of the 14 recommendations, four aligned with *Engage consumers*:

- Provide clear and simple messaging to patients that emphasize the expected benefits of PatientToc™ to patients
- Implement PatientToc™ first with specific patient sub-groups (e.g., complex patients)
- Ensure patients can complete PatientToc™ questionnaires within 2 to 10 min (e.g., pre-populate information when possible, reduce need for typing)
- Use PatientToc™ to optimize patient prescription wait times as well as other appointment-based services (e.g., MTM, medication synchronization program)

Accompanying implementation toolkit items included patient-facing print materials (large and small posters, pamphlets, bag stuffers), scripted language for pharmacies’ use, sample workflows, and workflow “cheat sheets.”

Three recommendations aligned with *Adapt and tailor to context*:

- Work with pharmacy teams and vendors to ensure PatientToc™ is well integrated with the pharmacies’ dispensing systems
- Consider adapting PatientToc™ for more languages (mockup/demo was provided in English only)
- Consider including patient education and/or information/referrals to pharmacy services on PatientToc™

These will primarily be addressed as part of the intervention development/finalized build of the adapted PatientToc™ application; however, a “Referrals Cheat Sheet” implementation toolkit item was created to provide a modifiable template of resources for pharmacy teams to consider when reviewing PatientToc™ results and addressing medication non-adherence. Of note, *Work with pharmacy teams and vendors to ensure PatientToc™ is well integrated with the pharmacies’ dispensing systems* was one of the most extensive adaptations to the PatientToc™ application and we plan to include this capability in the adapted finalized build during phase two.

## Discussion

Engaging key stakeholders from multiple perspectives provided invaluable insight into resources and strategies needed for initial implementation of PatientToc™ in community pharmacies. These collaborative efforts positioned us to prioritize actionable recommendations, refine and tailor an adapted PatientToc™ design, and develop an initial implementation toolkit unique to the community pharmacy context, which ultimately is expected to result in more effective spreading and scaling efforts. To our knowledge, this is the first study to evaluate electronic collection and use of electronic PRO data in community pharmacy settings using implementation science approaches. As stakeholders’ insights were the foundation of this pre-implementation evaluation, we focus our discussion on stakeholders’ highest priority recommendations. As well, we situate the findings in the implementation science and pharmacy practice literatures and discuss policy-related implications.

The first two high priority recommendations, (1) *provide clear and simple instructions to patients that emphasize the expected benefits of PatientToc™* and (2) *ensure that patients can complete PatientToc™ questionnaires within 2 to 10 min*, align with the ERIC implementation strategy “Engage Consumers: Prepare Patients/Consumers to be Active Participants” [15]. The first recommendation regarding the emphasis of expected benefits to patients has practice and policy-related implications. For example, in the US, medication therapy management (MTM) under Medicare Prescription Drug policy is a service often provided by community pharmacists [27]; however, the literature indicates lack of MTM uptake by some beneficiaries may be due to gaps in patient-friendly communication and marketing on MTM and its benefits [28]. Our toolkit items and specific strategies for this recommendation, including patient-facing print materials and scripted language for pharmacy team members, align with suggestions cited in the pharmacy practice literature [28] and could serve as a model for promoting adoption of other pharmacy services and related policies.

Similar to the second high priority recommendation of using more brief questionnaires, Rolstad et al. [29] review and meta-analysis findings suggested questionnaire response rates were statistically lower for longer questionnaires; however, the researchers argue that the impact of content should not be overlooked, further emphasizing the importance of buy-in of patients, regardless of questionnaire length. Our toolkit items and marketing and messaging strategies include benefits, specifically to patients, expected from completing PatientToc™ questionnaires. Time to complete the questionnaires will be examined during phase two (implementation) of this study.

The third and final high priority recommendation, *provide hands-on training and resources for pharmacy teams, possibly for continuing education credit, to support PatientToc™ implementation*, aligns with several ERIC implementation strategies in the “Train and Educate Stakeholders” category, including *develop and distribute education materials* and *work with educational institutions*. Our training toolkit items align with these strategies, particularly our Social Determinants of Health Continuing Education modules (per request of advisory panel members) offered in collaboration with a college of pharmacy continuing education office. This recommendation was not surprising, as implementation strategies focusing on training and educating stakeholders are ubiquitous. For example, Thoele et al. describe their intervention implementation process in an acute care hospital setting. Researchers developed 54 toolkit items over a three-phase process. Of these 54 toolkit items, 25 (46%) were related to training medical staff during the first two phases. Training and education strategies are often described as necessary for implementation efforts, but they are usually not sufficient on their own [30]; hence, our implementation toolkit is composed of a variety of implementation strategies spanning the nine ERIC implementation strategy categories.

Implementation science is an iterative process; thus, we are taking a similar approach and aim to do two plan-do-study-act cycles in the next phase of this study to refine our implementation toolkit for spreading and scaling PatientToc™ to community pharmacies. We anticipate revisions to the pharmacy team’s training modules may be required after initial implementation and prior to further scaling of PatientToc™.

### Limitations

While this study has several strengths, findings should be considered in the context of its limitations. First, only Midwestern and no chain community pharmacies were recruited in this phase of the study. However, we made great efforts to account for nuances in findings by pharmacy and participant type and we provide participating pharmacies’ demographic data, which can aid in transferring findings to different community pharmacy settings with similar characteristics. Nevertheless, we will attempt to recruit chain community pharmacies in the scale-up phase of evaluation and will revise implementation strategies as needed.

Although comprehensive, we applied a complex approach to data analysis by including all 41 constructs of CFIR, which ultimately resulted in over 200 inductive sub-codes. This approach was time consuming and required a change in our analysis approach from analysts independently coding and reconciling the same transcripts for all study sites to two sites with the remaining transcripts being divided across analysts to be independently coded with frequent research team member quality checks. Furthermore, results could have varied if a different implementation framework was used. However, CFIR proved to be useful as it addressed our study objectives, facilitated the identification of key implementation barriers and facilitators, and helped elicit recommendations, which corresponded to several widely recognized implementation strategies [15]. Lastly, the developmental formative evaluation approach used in this study prioritized qualitative methods to explore potential barriers, facilitators, and actionable recommendations for implementing PatientToc™ in community pharmacies. A more quantitative approach, such as a survey, could better capture frequencies of potential barriers, facilitators, and recommendations. However, using qualitative approaches, such as semi-structured interviews, was preferred for our goal of gaining in-depth perspectives from participants rather than a generalized understanding [31]. Thus, using both methods allowed access to data and interpretations of findings that each method alone could not provide.

## Conclusions

Adoption of health information technology, specifically to electronically capture PROs in the community pharmacy setting, is warranted. Applying implementation science methods and strategies can aid in adopting such interventions. If shown to be effective, the implementation strategies systematically developed in this study can serve as models for implementing other health information technology-driven, patient care services in the understudied context of community pharmacies.

## Supplementary Information

**Additional file 1.** Good Reporting of A Mixed Methods Study (GRAMMS) checklist.

**Additional file 2.** Data collection forms.

**Additional file 3.** Codebook.

## Data Availability

The datasets generated and/or analyzed during the current study are not publicly available because individuals’ privacy could be compromised. However, reasonable requests for deidentified datasets can be made to the corresponding author for consideration.

## Abbreviations

CAHPS®: Consumer Assessment of Healthcare Providers and Systems
CFIR: Consolidated Framework for Implementation Research
EBQI: Evidence-Based Quality Improvement
ERIC: Expert Recommendations for Implementing Change
GRAMMs: Good Reporting of a Mixed Methods Study
L.A.: Los Angeles
MTM: Medication therapy management
PROs: Patient-reported outcomes
REDCap: Research Electronic Data Capture
SD: Standard deviation
