# Supplementary material for: Preparing for the spread of patient-reported outcome (PRO) data collection from primary care to community pharmacy: a mixed-methods study
Source: Implement Sci Commun. 2022 Mar 14;3:29. doi: 10.1186/s43058-022-00277-3 (PMC8919161; doi:10.1186/s43058-022-00277-3)
Supplement: Supplementary file 1 — Additional file 1. Good Reporting of A Mixed Methods Study (GRAMMS) checklist. [file 43058_2022_277_MOESM1_ESM.docx]

**Good Reporting of A Mixed Methods Study (GRAMMS) checklist**

| **Guideline** | **Section: page** |
| --- | --- |
| Describe the justification for using a mixed methods approach to the research question | Methods: pg. 5 |
| Describe the design in terms of the purpose, priority and sequence of methods | Methods: pg. 5 |
| Describe each method in terms of sampling, data collection and analysis | Methods: pgs. 7 - 11 |
| Describe where integration has occurred, how it has occurred and who has participated in it | Methods: pg. 12 |
| Describe any limitation of one method associated with the presence of the other method | Discussion: pg. 21 |
| Describe any insights gained from mixing or integrating methods | Results: pgs. 15 – 16 Discussion: pgs. 21 |

O'Cathain A, Murphy E, Nicholl J. The quality of mixed methods studies in health services research. J Health Serv Res Policy. 2008;13: 92-98.
