# Supplementary material for: Preparing for the spread of patient-reported outcome (PRO) data collection from primary care to community pharmacy: a mixed-methods study
Source: Implement Sci Commun. 2022 Mar 14;3:29. doi: 10.1186/s43058-022-00277-3 (PMC8919161; doi:10.1186/s43058-022-00277-3)
Supplement: Supplementary file 2 — Additional file 2. Data collection forms. [file 43058_2022_277_MOESM2_ESM.docx]

Instructions & field notes template

**Observer:_________________**

**Dates Observed:_____________**

**Practice ID:__________**

**Demographic data to obtain once for each clinic/pharmacy from the site contact(s):**

**- Approximate date (for LA Net practices) when PatientToc implemented:______________**

**- Type of pharmacy/clinic (e.g., independent, chain, etc.)_________**

**- Number of provider/RPh FTEs________**

**- Number of staff/technician FTEs______**

**- Weekly Rx volume or patient clinic volume (total number of unique patients seen within a week (clinics))**

**- Tell me about how PatientToc is currently used at your facility (clinics)_________**

**- For pharmacies, summary of medication adherence services offered (e.g., delivery, auto refills, compliance packaging, med synch, participation in EQuIPP, mobile apps for patient use, 90 day fills, etc.)**

**- For pharmacies, summary of other patient care services offered (e.g., MTM, diabetes management, etc.)**

**- For pharmacies, a brief description of the pharmacy’s workflow (e.g., drive throughs?, walkup windows?)**

**General Notes (You will review/discuss aloud these during daily debriefs so we can audio-record and transcribe field notes for analysis)**

**Probes for “In the Field/contextual inquiry” interviews**

Note: Audio-record these conversations with permission and supplement with written notes below. On the recording, state your name, study ID number of participant, the task(s) being performed, and type of participant being interviewed.

At pharmacy sites, keep a PatientToc tablet with you in order to allow a participant to look at it/try it out when considering your questions.

**When to do these interviews:**

- When providers/staff (LA Net only) are preparing for the clinic day/use of PatientToc in clinic
- When patients (LA Net only) are working through questions on PatientToc
- When providers/staff (LA Net only) are reviewing patient responses in PatientToc
- When providers/staff (LA Net only) are writing up their visit notes/deciding on interventions
- When pharmacists/pharmacy staff are selecting patients in need of intervention, particularly for medication non-adherence
- When pharmacists/pharmacy staff are reviewing/considering patients flagged by contracted/employer programs (e.g., MTM vendors, internal programs embedded in dispensing software) as potentially non-adherent
- At other times when insight into relevant clinic/pharmacy workflow and the role of PatientToc implementation will be evident

**Probes:**

- What are you doing now?
- Why are you doing this?
- What alternatives did you consider for completing this task?
- What are barriers to getting this work done? Facilitators?
- What makes this work happen effectively? Efficiently?
- Is there a “secret sauce” for getting this work done?
- How, if at all, (for LA Net practices) has PatientToc made this harder or easier? Why is that?
- How, if at all, might (for pharmacy practices) PatientToc make this harder or easier? Why is that?
- If you could wave a wand and make something magically happen to improve this, what would it be?

Interview 1

Date/time: Study ID #: Task/summary of what is happening: Participant type:

Notes:

Interview 2

Date/time: Study ID #: Task/summary of what is happening: Participant type:

Notes:

Interview 3

Date/time: Study ID #: Task/summary of what is happening: Participant type:

Notes:

Interview 4

Date/time: Study ID #: Task/summary of what is happening: Participant type:

Notes:

Interview 5

Date/time: Study ID #: Task/summary of what is happening: Participant type:

Notes:

**Observation recording. Record your observations at the practice by answering the following questions.**

**(You will review these during daily debriefs so we can audio-record and transcribe field notes for analysis. State roles/study IDs; NOT names)**

**Intervention Characteristics**

Who decided (or would need to decide for a pharmacy) to implement PatientToc and why? [Intervention source]

What appears to be the sentiment re: the evidence behind PatientToc? [Evidence strength]

What, if any, advantage do stakeholders here think PatientToc has over alternatives? [Relative advantage]

What adjustments were made to make it work in this clinic? Would need to be made to make it work in a pharmacy? How easy/hard would this be? [Adaptability]

How easy/hard would it be to do PDSA cycles of PatientToc here (pharmacies)? How was PatientToc piloted? (clinics) [Trialability]

How complex would be/was PatientToc implementation? [Complexity]

How well is PatientToc “packaged” and presented to patients? [Design quality and packaging]

What considerations were made or would be needed with regards to costs of implementation? [Costs]

**Outer Setting**

What are unique patient needs at this site? How in tune are providers/staff with patient needs? [Patient needs and resources]

How well connected does the pharmacy/clinic seem to peer organizations/stakeholder groups? [Cosmopolitanism]

To what extent did/would peer pressure influence PatientToc implementation here? [Peer pressure]

What external policies (quality measures, regulations, etc.) have/would influence PatientToc implementation here? [External policy and incentives]

**Inner Setting**

How is the pharmacy/clinic organized? How long has it been around? How did or would the infrastructure of the clinic/pharmacy influence PatientToc implementation? [Structural characteristics]

How well do staff appear to be communicate? [Networks and communications]

What is the culture of the organization? [Culture]

What is the implementation climate? [Implementation climate]

Do people here feel change was/is needed? Why? [Tension for change]

How well does PatientToc seem to “fit” here? [Compatibility]

How important do people think it is/was to implement PatientToc? [Relative priority]

What rewards or incentives has the clinic associated with PatientToc or do you think the pharmacy would use to encourage adoption by staff? [Organizational incentives and rewards]

What goals have been set (clinics) or would likely be set (pharmacies) related to PatientToc and does/would feedback on goals occur? [Goals and feedback]

How well/not well does the organization climate support learning? [Learning climate]

What level of endorsement or support for PatientToc has been observed? [Readiness for implementation-Leadership engagement]

What resources were/would be available for implementation? [Readiness for implementation-Available resources]

What training was provided/would be needed about PatientToc implementation? [Access to knowledge and information]

**Characteristics of Individuals**

How do people feel about PatientToc? Is there variation/differing opinions? (State roles/study IDs; NOT names) [Knowledge and beliefs about the intervention]

How confident were/are people in their ability to implement PatientToc? Is there variation/differing opinions(State roles/study IDs; NOT names) [Self-efficacy]

How ready were/are participants to implement PatientToc? Is there variation/differing opinions(State roles/study IDs; NOT names) [Individual stage of change]

How have/would it appear that individuals’ relationship/feelings about the clinic/pharmacy influence PatientToc implementation? Is there variation/differing opinions? (State roles/study IDs; NOT names) [Individual identification with organization]

How have/would other personal attributes observed (e.g., motivation, values, competence, etc.) influence PatientToc implementation? (State roles/study IDs; NOT names) [Other personal attributes]

**Process**

What was the implementation plan or what might it need to look like for this pharmacy? [Planning]

Who were/are the key individuals to engage? (State roles/study IDs; NOT names) [Engaging-Opinion leaders]

How led/would be anticipated to lead implementation? (State roles/study IDs; NOT names) [Engaging-Formally appointed internal implementation leaders]

Other than the formal leader, who did/would be expected to champion for PatientToc? (State roles/study IDs; NOT names) [Engaging-Champions)

Did a team outside the clinic help with implementation? How did this go? OR What would an external implementation team need to consider at this pharmacy? [Engaging-External change agents]

What steps have/would be needed to be taken to encourage people to commit to using PatientToc? [Engaging-Key stakeholders]

What is (what should it be) patient communication/workflow for PatientToc like? [Engaging-Intervention participants]

How has implementation gone? [Executing]

What information has/should be collected to help with evaluating PatientToc implementation? [Reflecting and evaluating]

**Additional Notes**

Interview guide: L.A. Net Providers (i.e., locations where PatientToc WAS implemented)

Study ID#: ________________

***Below are draft script/interview guide/demographic items for the semi-structured interviews. These will be piloted with a small number of providers and minor modifications and/or deletions/additions may be made prior to/throughout data collection to ensure items are clear, as relevant as possible, and comprehensive.***

**Opening script:**

As a reminder, I’m going to ask you about your experiences and/or ideas related to PatientToc implementation. Please remember that there are no right or wrong answers; we just want your opinion. Your responses will be confidential and only de-identified information will be shared. Please don’t state any names on the recording. Do you have any questions before I turn on the recorder and we get started?

To begin, please tell me about how you use PatientToc. What was most helpful in making PatientToc work in your clinic. What were some of the challenges?

*[Interviewer should then proceed to questions from the below list, organized by CFIR domain/construct, to probe on constructs not addressed or only partially addressed by participant in their summary. It is expected that not all questions will be asked.]*

Examples of questions planned to be loaded into PatientToc for future implementation in community pharmacies. Interviewer should refer to these and tablet to help describe the planned intervention:

3. [From the BMQ] How much problem or concern are you having in the following areas [circle one]

None A little A lot

a. My medication causes side effects. . . . . . . . . . . . . . . . . . . . . . . . . . 0 1 2

b.It is hard to remember all the doses . . . . . . . . . . . . . . . . . . . . . . . . . 0 1 2

c. It is hard to pay for the medication . . . . . . . . . . . . . . . . . . . . . . . . . 0 1 2

d.It is hard to open the container. . . . . . . . . . . . . . . . . . . . . . . . . . . . . 0 1 2

e. It is hard to get my refill on time . . . . . . . . . . . . . . . . . . . . . . . . . . . 0 1 2

f. It is hard to read the print on the container . . . . . . . . . . . . . . . . . . . 0 1 2

g. The dosage times are inconvenient . . . . . . . . . . . . . . . . . . . . . . . . . 0 1 2

h. My medication causes other problem or concern . . . . . . . . . . . . . . . 0 1 2

If other problem or concern, please explain: ________________________________________________________________

| **CFIR Domain/Construct/Sub-Construct** | **Questions & Probes** |
| --- | --- |
| **I. Intervention Characteristics** |  |
| **A. Intervention Source** | How was the decision to implement PatientToc™ at your clinic made? Who made the decision? What did you think about it? |
| **B. Evidence Strength & Quality** | What kind of information or evidence are you aware of that shows whether PatientToc has worked in your clinic? What do clinic leaders think of PatientToc? How, if at all, does this information affect your perception of PatientToc? What role, if any, did this information play in determining “roll out” of PatientToc across LA Net clinics? |
| **C. Relative Advantage** | How does PatientToc™ compare to other alternatives that may have been considered or used previously? What advantages does it have? Disadvantages? Are there other interventions you believe would be more beneficial? Why is that? |
| **D. Adaptability** | What kind of changes or alterations were made to PatientToc™ to make it work in your clinic? How were these decisions made? |
| **E. Trialability** | To what extent was PatientToc piloted prior to full-scale implementation at your clinic? What adjustments were made to PatientToc™ prior to its full-scale implementation at your clinic? How effective was the trial run for PatientToc™? Why is that? |
| **F. Complexity** | How complicated is it to use PatientToc™ in your clinic? Describe your experience with PatientToc with regards to how user friendly it is and how it fits in your workflow. How complicated or not complicated do you think it would be for patients to answer these types of questions [researcher refers to examples from the BMQ, above] in PatientToc when visiting a pharmacy? Why do you say that? What changes, if any, would be needed to make it less complicated? About how much time do you think it would take for patients to answer questions like this? |
| **G. Design Quality & Packaging** | What resources or toolkits, if any, were or are you exposed to in order to help implement PatientToc™ in your setting? What other resources, if any, might be ideal? If you were to teach implementation to another colleague, what would you need to help you? What design features of PatientToc help or hinder its use? |
| **H. Costs** | What costs were considered and/or measured when deciding to implement PatientToc™ in your setting? |
| **II. Outer Setting** |  |
| **A. Patient Needs & Resources** | To what extent were patient needs and preferences considered when deciding to implement PatientToc? How have patients responded to PatientToc? Any barriers to use? Examples? What alterations were made to meet specific needs/preferences? What additional alterations, if any, might be needed? Why? Examples of things patients have said about PatientToc? |
| **B. Cosmopolitimism** | What kind of information exchange/discussions do you have with others outside your setting, related to PatientToc? |
| **C. Peer Pressure** | To what extent does implementing PatentToc provide an advantage for your organization compared to other organizations in your area? Examples? |
| **D. External Policies & Incentives** | What kind of local, state, or national performance measures, policies, regulations, or guidelines influenced the decision to implement PatientToc? Why? How has your organization’s performance measures, policies, regulations, or guidelines been affected by PatientToc? |
| **III. Inner Setting** |  |
| **A. Structural Characteristics** | How did the infrastructure of your organization (social architecture, age, maturity, size, or physical layout) affect the implementation of PatientToc? How did the infrastructure facilitate/hinder implementation? Examples? What kind of infrastructure changes were needed? What did this process entail? |
| **B. Networks & Communication** | How do you typically find out about new information, such as new initiatives like PatientToc? How did communication about PatientToc go? |
| **C. Culture** | How do you think your organization's culture (general beliefs, values, assumptions that people embrace) affected the implementation of the intervention? To what extent are new ideas embraced and used to make improvements in your organization? Can you describe a recent example? |
| **D. Implementation Climate** | What was the general level of receptivity in your organization to implementing PatientToc? Why? |
| **1. Tension for Change** | Was there a strong need for PatientToc? Why or why not? Do you think others at your organization would agree? Why/why not? |
| **2. Compatibility** | How well does PatientToc align with your values and norms and the values and norms within the organization? How well did PatientToc fit with existing work processes and practices in your setting? How was PatientToc integrated into existing processes? |
| **3. Relative Priority** | What was the priority of getting PatientToc implemented relative to other initiatives that were happening at the time? What influenced prioritization? How were competing demands juggled? |
| **4. Organizational Incentives & Rewards** | What kinds of incentives were in place to help ensure that the implementation PatientToc was successful? What was your personal motivation for ensuring success? |
| **5. Goals & Feedback** | To what extent has your organization set goals related to the implementation PatientToc? Please describe and give examples. How does PatientToc align with other organizational goals? |
| **6. Learning Climate** | To what extent do you feel like you can try new things to improve your work processes? Why is that? |
| **A. Readiness for Implementation** | *[Addressed through sub-constructs below]* |
| **1. Leadership Engagement** | What level of endorsement or support for PatientToc have you seen or heard from senior leaders at your organization? What role have they had in implementation? How did this help/hinder the process? |
| **2. Available Resources** | To what extent did you have sufficient resources to implement PatientToc? Would you have wanted other resources? Give examples. |
| **3. Access to Knowledge & Information** | What kind of training about PatientToc did you receive? How did this go? What, if anything, could have been improved? Who do you ask if you have questions about PatientToc? |
| **IV. Characteristics of Individuals** |  |
| **A. Knowledge & Beliefs about the Intervention** | How do you think use of PatientToc is going (or went, if no longer used)? Why do you say that? |
| **B. Self-Efficacy** | How confident or not confident are you in your use of PatientToc? Why? |
| **C. Individual Stage of Change** | How prepared did you feel to use PatientToc in the beginning? |
| **D. Individual Identification with Organization** | *[Will evaluate/code based on other responses]* |
| **E. Other Personal Attributes** | *[Will evaluate/code based on other responses]* |
| **V. Process** |  |
| **A. Planning** | Can you describe the plan that was used for implementing PatientToc? What was your role in the planning process? How did this go? |
| **B. Engaging** | *[Assessed by sub-constructs below]* |
| **1. Opinion Leaders** | Who were the key influential individuals to get on board with PatientToc? |
| **2. Formally Appointed Internal Implementation Leaders** | Who led implementation of PatientToc? *[Just describe their role; not an actual name]* How did/will this person come into this role? Appointed? Volunteered? Voluntold? How did the selection of this individual affect implementation? How might implementation have gone differently had another individual led it? Who else is involved with leading the implementation? |
| **3. Champions** | Other than the formal implementation leader, are there people in your organization who championed (go above and beyond what might be expected) PatientToc? How did this come about? |
| **4. External Change Agents** | Did someone (or a team) outside your organization help you with implementing the intervention? What worked or didn’t work? |
| **5. Key Stakeholders** | What steps have been taken to encourage individuals to commit to using PatientToc? Who are the key individuals to engage? How does word get out to them? |
| **6. Intervention Participants** | How did/do you or your colleagues communicate to patients about PatientToc? What does patient workflow with PatientToc look like? |
| **C. Executing** | To what extent has PatientToc been implemented according to the implementation plan? Why is that? |
| **D. Reflecting & Evaluating** | What information has been collected to evaluate PatientToc implementation? What, if any, feedback has been elicited from patients? |

**Wrap up questions:**

-Anything we haven’t talked about that we need to cover?

-Any closing thought or recommendations for us?

**Demographic Data to Collect Verbally Following the Interview**

**All numbering will start with “provider” and then the number**

1. Age: ___________ years

□ Prefer not to answer_999_

2. Gender

□ Male_1_

□ Female_2_

□ Prefer not to answer_3_

3. Ethnicity

□ Hispanic/Latino_1_

□ Not Hispanic/Latino_0_

□ Prefer not to answer_2_

4. Race

□ White/Caucasion_1_

□ Black/African American_2_

□ Asian_3_

□ American Indian/Alaska Native_4_

□ Native Hawaiian or Other Pacific Islander_5_

□ More than one race_6_

□ Prefer not to answer_7_

5. Type of provider

□ Physician (MD, DO)_1_

□ Physician Assistant_2_

□ Nurse Practitioner_3_

□ Other_4_ (please specify)__________

6. Year completed terminal degree and/or clinical training:____________

7. Specialty:____________________

8. Year employment began at THIS clinic: _____________

9. Approximate percent of hours per week working directly with patients: ______________%

10. Approximate number of patients seen in a typical week:___________

11. Date began using PatientToc:________/_________

12. Approximate number of patients PatientToc is used with in a typical week:_________________%

Interview guide: L.A. Net Staff (i.e., locations where PatientToc WAS implemented)

Study ID#: ________________

**Opening script:**

As a reminder, I’m going to ask you about your experiences and/or ideas related to PatientToc implementation. Please remember that there are no right or wrong answers; we just want your opinion. Your responses will be kept confidential and only de-identified information will be shared. Please don’t state any names on the recording. Do you have any questions before I turn on the recorder and we get started?

To begin, please tell me about how you use PatientToc. To begin, please tell me about how you use PatientToc. What was most helpful in making PatientToc work in your clinic. What were some of the challenges?

*[Interviewer should then proceed to questions from the below list, organized by CFIR domain/construct, to probe on constructs not addressed or only partially addressed by participant in their summary. It is expected that not all questions will be asked.]*

Examples of questions planned to be loaded into PatientToc for future implementation in community pharmacies. Interviewer should refer to these and tablet to help describe the planned intervention:

3. [From the BMQ] How much problem or concern are you having in the following areas [circle one]

None A little A lot

a. My medication causes side effects. . . . . . . . . . . . . . . . . . . . . . . . . . 0 1 2

b.It is hard to remember all the doses . . . . . . . . . . . . . . . . . . . . . . . . . 0 1 2

c. It is hard to pay for the medication . . . . . . . . . . . . . . . . . . . . . . . . . 0 1 2

d.It is hard to open the container. . . . . . . . . . . . . . . . . . . . . . . . . . . . . 0 1 2

e. It is hard to get my refill on time . . . . . . . . . . . . . . . . . . . . . . . . . . . 0 1 2

f. It is hard to read the print on the container . . . . . . . . . . . . . . . . . . . 0 1 2

g. The dosage times are inconvenient . . . . . . . . . . . . . . . . . . . . . . . . . 0 1 2

h. My medication causes other problem or concern . . . . . . . . . . . . . . . 0 1 2

If other problem or concern, please explain: ________________________________________________________________

| **CFIR Domain/Construct/Sub-Construct** | **Questions & Probes** |
| --- | --- |
| **I. Intervention Characteristics** |  |
| **A. Intervention Source** | How was the decision to implement PatientToc™ at your clinic made? Who made the decision? What did you think about it? |
| **B. Evidence Strength & Quality** | What kind of information or evidence are you aware of that shows whether PatientToc has worked in your clinic? What do clinic leaders think of PatientToc? How, if at all, does this information affect your perception of PatientToc? What role, if any, did this information play in determining “roll out” of PatientToc across LA Net clinics? |
| **C. Relative Advantage** | How does PatientToc™ compare to other alternatives that may have been considered or used previously? What advantages does it have? Disadvantages? Are there other interventions you believe would be more beneficial? Why is that? |
| **D. Adaptability** | What kind of changes or alterations were made to PatientToc™ to make it work in your clinic? How were these decisions made? |
| **E. Trialability** | To what extent was PatientToc piloted prior to full-scale implementation at your clinic? What adjustments were made to PatientToc™ prior to its full-scale implementation at your clinic? How effective was the trial run for PatientToc™? Why is that? |
| **F. Complexity** | How complicated is it to use PatientToc™ in your clinic? Describe your experience with PatientToc with regards to how user friendly it is and how it fits in your workflow. How complicated or not complicated do you think it would be for patients to answer these types of questions [researcher refers to examples from the BMQ, above] in PatientToc when visiting a pharmacy? Why do you say that? What changes, if any, would be needed to make it less complicated? About how much time do you think it would take for patients to answer questions like this? |
| **G. Design Quality & Packaging** | What resources or toolkits, if any, were or are you exposed to in order to help implement PatientToc™ in your setting? What other resources, if any, might be ideal? If you were to teach implementation to another colleague, what would you need to help you? What design features of PatientToc help or hinder its use? |
| **H. Costs** | What costs were considered and/or measured when deciding to implement PatientToc™ in your setting? |
| **II. Outer Setting** |  |
| **A. Patient Needs & Resources** | To what extent were patient needs and preferences considered when deciding to implement PatientToc? How have patients responded to PatientToc? Any barriers to use? Examples? What alterations were made to meet specific needs/preferences? What additional alterations, if any, might be needed? Why? Examples of things patients have said about PatientToc? |
| **B. Cosmopolitanism** | What kind of information exchange/discussions do you have with others outside your setting, related to PatientToc? |
| **C. Peer Pressure** | To what extent does implementing PatentToc provide an advantage for your organization compared to other organizations in your area? Examples? |
| **D. External Policies & Incentives** | What kind of local, state, or national performance measures, policies, regulations, or guidelines influenced the decision to implement PatientToc? Why? How has your organization’s performance measures, policies, regulations, or guidelines been affected by PatientToc? |
| **III. Inner Setting** |  |
| **A. Structural Characteristics** | How did the infrastructure of your organization (social architecture, age, maturity, size, or physical layout) affect the implementation of PatientToc? How did the infrastructure facilitate/hinder implementation? Examples? What kind of infrastructure changes were needed? What did this process entail? |
| **B. Networks & Communication** | How do you typically find out about new information, such as new initiatives like PatientToc? How did communication about PatientToc go? |
| **C. Culture** | How do you think your organization's culture (general beliefs, values, assumptions that people embrace) affected the implementation of the intervention? To what extent are new ideas embraced and used to make improvements in your organization? Can you describe a recent example? |
| **D. Implementation Climate** | What was the general level of receptivity in your organization to implementing PatientToc? Why? |
| **1. Tension for Change** | Was there a strong need for PatientToc? Why or why not? Do you think others at your organization would agree? Why/why not? |
| **2. Compatibility** | How well does PatientToc align with your values and norms and the values and norms within the organization? How well did PatientToc fit with existing work processes and practices in your setting? How was PatientToc integrated into existing processes? |
| **3. Relative Priority** | What was the priority of getting PatientToc implemented relative to other initiatives that were happening at the time? What influenced prioritization? How were competing demands juggled? |
| **4. Organizational Incentives & Rewards** | What kinds of incentives were in place to help ensure that the implementation PatientToc was successful? What was your personal motivation for ensuring success? |
| **5. Goals & Feedback** | To what extent has your organization set goals related to the implementation PatientToc? Please describe and give examples. How does PatientToc align with other organizational goals? |
| **6. Learning Climate** | To what extent do you feel like you can try new things to improve your work processes? Why is that? |
| **A. Readiness for Implementation** | *[Addressed through sub-constructs below]* |
| **1. Leadership Engagement** | What level of endorsement or support for PatientToc have you seen or heard from senior leaders at your organization? What role have they had in implementation? How did this help/hinder the process? |
| **2. Available Resources** | To what extent did you have sufficient resources to implement PatientToc? Would you have wanted other resources? Give examples. |
| **3. Access to Knowledge & Information** | What kind of training about PatientToc did you receive? How did this go? What, if anything, could have been improved? Who do you ask if you have questions about PatientToc? |
| **IV. Characteristics of Individuals** |  |
| **A. Knowledge & Beliefs about the Intervention** | How do you think use of PatientToc is going (or went, if no longer used)? Why do you say that? |
| **B. Self-Efficacy** | How confident or not confident are you in your use of PatientToc? Why? |
| **C. Individual Stage of Change** | How prepared did you feel to use PatientToc in the beginning? |
| **D. Individual Identification with Organization** | *[Will evaluate/code based on other responses]* |
| **E. Other Personal Attributes** | *[Will evaluate/code based on other responses]* |
| **V. Process** |  |
| **A. Planning** | Can you describe the plan that was used for implementing PatientToc? What was your role in the planning process? How did this go? |
| **B. Engaging** | *[Assessed by sub-constructs below]* |
| **1. Opinion Leaders** | Who were the key influential individuals to get on board with PatientToc? |
| **2. Formally Appointed Internal Implementation Leaders** | Who led implementation of PatientToc? *[Just describe their role; not an actual name]* How did/will this person come into this role? Appointed? Volunteered? Voluntold? How did the selection of this individual affect implementation? How might implementation have gone differently had another individual led it? Who else is involved with leading the implementation? |
| **3. Champions** | Other than the formal implementation leader, are there people in your organization who championed (go above and beyond what might be expected) PatientToc? How did this come about? |
| **4. External Change Agents** | Did someone (or a team) outside your organization help you with implementing the intervention? What worked or didn’t work? |
| **5. Key Stakeholders** | What steps have been taken to encourage individuals to commit to using PatientToc? Who are the key individuals to engage? How does word get out to them? |
| **6. Intervention Participants** | How did/do you or your colleagues communicate to patients about PatientToc? What does patient workflow with PatientToc look like? |
| **C. Executing** | To what extent has PatientToc been implemented according to the implementation plan? Why is that? |
| **D. Reflecting & Evaluating** | What information has been collected to evaluate PatientToc implementation? What, if any, feedback has been elicited from patients? |

**Wrap up questions:**

-Anything we haven’t talked about that we need to cover?

-Any closing thought or recommendations for us?

**Demographic Data to Collect Verbally Following the Interview:**

1. Age: ___________ years

□ Prefer not to answer_999_

2. Gender

□ Male_1_

□ Female_2_

□ Prefer not to answer_3_

3. Ethnicity

□ Hispanic/Latino_1_

□ Not Hispanic/Latino_0_

□ Prefer not to answer_2_

4. Race

□ White/Caucasion_1_

□ Black/African American_2_

□ Asian_3_

□ American Indian/Alaska Native_4_

□ Native Hawaiian or Other Pacific Islander_5_

□ More than one race_6_

□ Prefer not to answer_7_

5. Type of staff

□ Receptionist/front office_1_

□ Medical Assistant_2_

□ Nurse_3_

□ Other_4_ (please specify)__________

6. Year completed terminal degree and/or training to work in a clinic:____________

7. Year employment began at THIS clinic: _____________

8. Approximate percent of working hours per week spent working directly with patients: ______________%

9. Date began using PatientToc:______/________

10. Approximate number of unique patients seen in a typical week.___________

11. Approximate number of patients PatientToc is used with in a typical week:_________________%

Study ID#: ________________

***[Note: Below are draft script/interview guide/demographic items for the semi-structured interviews. These will be piloted with a small number of patients and minor modifications and/or deletions/additions may be made prior to data collection to ensure items are clear, as relevant as possible, and comprehensive.]***

**Opening script:**

As a reminder, I’m going to ask you about your experiences and/or ideas related to PatientToc implementation. Please remember that there are no right or wrong answers; we just want your opinion. Your responses will be kept confidential and only information where your name has been removed will be shared. Please don’t state any names on the recording. Do you have any questions before I turn on the recorder and we get started?

To begin, Could you tell me a little bit about the last time you used the tablet (PatientToc…mirror subject’s terminology)

Probes: when was that, what types of things did you do on it? what types of questions did you answer?

*[Interviewer should then proceed to questions from the below list, organized by CFIR domain/construct, to probe on constructs not addressed or only partially addressed by participant in their summary. It is expected that not all questions will be asked.]*

Examples of questions planned to be loaded into PatientToc for future implementation in community pharmacies. Interviewer should refer to these and tablet to help describe the planned intervention:

3. [From the BMQ] How much problem or concern are you having in the following areas [circle one]

None A little A lot

a. My medication causes side effects. . . . . . . . . . . . . . . . . . . . . . . . . . 0 1 2

b.It is hard to remember all the doses . . . . . . . . . . . . . . . . . . . . . . . . . 0 1 2

c. It is hard to pay for the medication . . . . . . . . . . . . . . . . . . . . . . . . . 0 1 2

d.It is hard to open the container. . . . . . . . . . . . . . . . . . . . . . . . . . . . . 0 1 2

e. It is hard to get my refill on time . . . . . . . . . . . . . . . . . . . . . . . . . . . 0 1 2

f. It is hard to read the print on the container . . . . . . . . . . . . . . . . . . . 0 1 2

g. The dosage times are inconvenient . . . . . . . . . . . . . . . . . . . . . . . . . 0 1 2

h. My medication causes other problem or concern . . . . . . . . . . . . . . . 0 1 2

If other problem or concern, please explain: ________________________________________________________________

| **CFIR Domain/Construct/Sub-Construct** | **Questions & Probes** |
| --- | --- |
| **B. Evidence Strength & Quality** | How important or not important do you feel using PatientToc at this clinic is? Why is that? What information could the clinic/doctor provide about PatientToc that would change your opinion? |
| **C. Relative Advantage** | How does PatientToc™ compare to other similar technologies that you may have used previously for collecting information form patients? What advantages does it have? Disadvantages? Are there other interventions you believe would be more beneficial to patients? Why is that? |
| **F. Complexity** | How hard is it to use PatientToc™? How easy is it? About how much time does it take you to complete the questions? What helps or does not help to make it go quicker?  What changes do you think would need to happen to me able to use the tablets (PatientToc) at your pharmacy to answer questions about your medications. How easy or hard do you think it would be to answer these types of questions [BMQ listed above] in the tablet when visiting your pharmacy? What changes if any would make it easier? |
| **G. Design Quality & Packaging** | What design features of PatientToc help or hinder its use? |
| **A. Patient Needs & Resources** | What are your main types of questions, concerns, and preferences when you are visiting your doctor? How aware or unaware are your doctors and their staff of your questions, concerns, and preferences ? How well or not well are your needs met using PatientToc |
| **A. Knowledge & Beliefs about the Intervention** | What do you think about PatientToc? How do you think use of PatientToc is going? Why do you say that?  What benefits/ problems do you think there are from using the tablets at this clinic? |
| **B. Self-Efficacy** | How confident or not confident are you in your use of PatientToc? Why? |
| **6. Intervention Participants** | How did the doctor’s office first introduce you to/ show you how to use PatientToc? Walk me through your appointment at this clinic. Tell me when/how PatientToc is used throughout your clinic visit. |
| **D. Reflecting & Evaluating** | Outside of this conversation, has this clinic asked for any feedback about PatientToc? Tell me about the type of feedback that you have been asked for/ given the clinic about PatientToc? |

**Wrap up questions:**

-Anything we haven’t talked about that we need to cover?

-Any closing thought or recommendations for us?

**Demographic Data to Collect Verbally Following the Interview:**

1. Age: ___________ years

2. Gender

□ Male_1_

□ Female_2_

□ Prefer not to answer_3_

3. Ethnicity

□ Hispanic/Latino_1_

□ Not Hispanic/Latino_0_

□ Prefer not to answer_2_

4. Race

□ White/Caucasion_1_

□ Black/African American_2_

□ Asian_3_

□ American Indian/Alaska Native_4_

□ Native Hawaiian or Other Pacific Islander_5_

□ More than one race_6_

□ Prefer not to answer_7_

5. In a typical year, about how often to do you visit THIS doctor’s office? _______________

6. How many different chronic conditions do you have that require routine prescription medication use? You do not need to specify which conditions___________

If you aren't sure what a chronic condition is, here are some examples:

Hypertension (High blood pressure)

Hyperlipidemia (High cholesterol)

Diabetes

Heart Failure

Atrial Fibrillation

Arthritis (Osteoarthritis and Rheumatoid)

Hepatitis (Chronic Viral B & C)

Cirrhosis or other liver disease

Depression

Asthma

HIV/AIDS

Autism Spectrum Disorders or ADD/ADHD

Narcolepsy or other sleep/wake disorder

Cancer (Breast, Lung, and Prostate)

Chronic pain

Irritable Bowel Syndrome

Inflammatory Bowel Disease (Crohn's Disease or

ulcerative colitis)

Lupus or other autoimmune disease

Thyroid disorder

Epilepsy or other seizure disorder

Hypothyroidism or hyperthyroidism

Alzheimer's Disease and related Dementia

Substance-use disorder

Psoriasis or other chronic skin condition

7. How many different_______medications do you use regularly?

Prescription___________

Non-prescription/OTC___________

Herbals/supplements____________

8. Approximate Date you began using PatientToc:______/________

9. Of the [number of visits answered in question 5] visits, to THIS doctor’s office, how many times was PatientToc used?

10. Is English the subject’s primary language? Yes/No (investigator to answer)

11. Was a translator used during this interview? Yes/No (investigator to answer)

Interview guide: Pharmacists (i.e., locations where PatientToc NOT implemented)

Study ID#: ________________

**Opening script:**

As a reminder, I’m going to ask you about your ideas related to PatientToc implementation. Please remember that there are no right or wrong answers; we just want your opinion. Your responses will be confidential and only information with your name removed will be shared. Please don’t state any names on the recording. Do you have any questions before I turn on the recorder and we get started?

To begin, please tell me your initial thoughts about PatientToc now that you have seen and been able to spend a few minutes with a tablet.

- How might you approach implementing PT in your pharmacy?
- What kinds of problems might you expect in trying to implement?
- What about PT or how your pharmacy already operates might help with the implementation?
- At this point, do you see anything that might need to change about PT or your workflow to make this work?

*[Interviewer should then proceed to questions from the below list, organized by CFIR domain/construct, to probe on constructs not addressed or only partially addressed by participant in their summary. It is expected that not all questions will be asked.]*

Examples of questions planned to be loaded into PatientToc for future implementation in community pharmacies. Interviewer should refer to these and tablet to help describe the planned intervention:

3. [From the BMQ] How much problem or concern are you having in the following areas [circle one]

None A little A lot

a. My medication causes side effects. . . . . . . . . . . . . . . . . . . . . . . . . . 0 1 2

b.It is hard to remember all the doses . . . . . . . . . . . . . . . . . . . . . . . . . 0 1 2

c. It is hard to pay for the medication . . . . . . . . . . . . . . . . . . . . . . . . . 0 1 2

d.It is hard to open the container. . . . . . . . . . . . . . . . . . . . . . . . . . . . . 0 1 2

e. It is hard to get my refill on time . . . . . . . . . . . . . . . . . . . . . . . . . . . 0 1 2

f. It is hard to read the print on the container . . . . . . . . . . . . . . . . . . . 0 1 2

g. The dosage times are inconvenient . . . . . . . . . . . . . . . . . . . . . . . . . 0 1 2

h. My medication causes other problem or concern . . . . . . . . . . . . . . . 0 1 2

If other problem or concern, please explain: ________________________________________________________________

| **CFIR Domain/Construct/Sub-Construct** | **Questions & Probes** |
| --- | --- |
| **I. Intervention Characteristics** |  |
| **A. Intervention Source** | For PatientToc™ to be implemented at your pharmacy, who would need to make the decision? What would you think about the possibility of implementation? |
| **B. Evidence Strength & Quality** | What kind of information or evidence would be needed for you to think PatientToc could work in your pharmacy? |
| **C. Relative Advantage** | How does PatientToc™ compare to other alternatives that may have been considered or used previously? What advantages does it have? Disadvantages? Are there other interventions you believe would be more beneficial? Why is that? |
| **D. Adaptability** | What kind of changes or alterations to PatientToc™ would be needed to make it work in your pharmacy? Why is that? |
| **E. Trialability** | Why types of PatientToc piloting would be needed prior to full-scale implementation at your pharmacy? Why is that? |
| **F. Complexity** | How complicated do you think it would be to use PatientToc™ in your pharmacy? How complicated or not complicated do you think it would be for patients to answer these types of questions [researcher refers to examples from the BMQ, above] in PatientToc when visiting your pharmacy? Why do you say that? What changes, if any, would be needed to make it less complicated? About how much time do you think it would take patients to answer questions like this? How likely or unlikely do you think it would be for patients to have that much time? What would help or not help? How, if it all, do you think the patient “flow” during pharmacy visits might need to change for it to work? |
| **G. Design Quality & Packaging** | What resources or toolkits, if any, would you want to help implement PatientToc™ in your setting? What other resources, if any, might be ideal? |
| **H. Costs** | What costs would you want to consider and/or measure when deciding to implement PatientToc™ in your setting? |
| **II. Outer Setting** |  |
| **A. Patient Needs & Resources** | How do you think patients would respond to PatientToc? Any barriers to use? Examples? What alterations do you think would be needed made to meet specific needs/preferences? |
| **B. Cosmopolitanism** | What kind of information exchange/discussions would you anticipate having with others outside your setting, related to PatientToc if it were implemented? |
| **C. Peer Pressure** | Can you tell me what you know about any other organizations that have implemented PatientToc or other similar programs? To what extent would implementing PatentToc provide an advantage for your organization compared to other organizations in your area? Examples? |
| **D. External Policies & Incentives** | What kind of local, state, or national performance measures, policies, regulations, or guidelines might influence the decision to implement PatientToc? Why? |
| **III. Inner Setting** |  |
| **A. Structural Characteristics** | How would the infrastructure of your organization (social architecture, age, maturity, size, or physical layout) affect the implementation of PatientToc? How would the infrastructure facilitate/hinder implementation? Examples? What kind of infrastructure changes would be needed? What would this process entail? |
| **B. Networks & Communication** | How do you typically find out about new information, such as new initiatives like PatientToc if it were implemented? |
| **C. Culture** | How do you think your organization's culture (general beliefs, values, assumptions that people embrace) would affect the implementation of PatientToc? To what extent are new ideas embraced and used to make improvements in your organization? Can you describe a recent example? |
| **D. Implementation Climate** | What would you expect the general level of receptivity in your organization to implementing PatientToc? Why? |
| **1. Tension for Change** | Is there a strong need for PatientToc? Why or why not? Do you think others at your organization would agree? Why/why not? |
| **2. Compatibility** | How well would PatientToc fit with existing work processes and practices in your setting? How would PatientToc be integrated into existing processes? |
| **3. Relative Priority** | How would you prioritize getting PatientToc implemented relative to other initiatives that are happening? Why is that? |
| **4. Organizational Incentives & Rewards** | What kinds of incentives would be needed to help ensure that the implementation PatientToc is successful? What would be your personal motivation for ensuring success? |
| **5. Goals & Feedback** | How does PatientToc align with goals of the pharmacy? |
| **6. Learning Climate** | To what extent do you feel like you can try new things to improve your work processes? Why is that? |
| **A. Readiness for Implementation** | *[Addressed through sub-constructs below]* |
| **1. Leadership Engagement** | What level of endorsement or support for PatientToc would you anticipate from senior leaders at your organization? |
| **2. Available Resources** | What resources would be needed to successfully implement PatientToc? |
| **3. Access to Knowledge & Information** | What kind of training about PatientToc would you need to receive? |
| **IV. Characteristics of Individuals** |  |
| **A. Knowledge & Beliefs about the Intervention** | *[Will evaluate/code based on other responses]* |
| **B. Self-Efficacy** | *[Will evaluate/code based on other responses]* |
| **C. Individual Stage of Change** | How prepared do you feel to use PatientToc? Why? |
| **D. Individual Identification with Organization** | *[Will evaluate/code based on other responses]* |
| **E. Other Personal Attributes** | *[Will evaluate/code based on other responses]* |
| **V. Process** |  |
| **A. Planning** | What plan would you suggest for implementing PatientToc? |
| **B. Engaging** | *[Assessed by sub-constructs below]* |
| **1. Opinion Leaders** | Who would be the key influential individuals to get on board with PatientToc? |
| **2. Formally Appointed Internal Implementation Leaders** | Who would you anticipate leading implementation of PatientToc? *[Just describe their role; not an actual name]* How would you anticipate this person coming into this role? Appointed? Volunteered? Voluntold? How might implementation go differently if another individual led it? Who else do you think would be involved with leading the implementation? |
| **3. Champions** | Other than the formal implementation leader, are there people in your organization who you think would champion (go above and beyond what might be expected) PatientToc? |
| **4. External Change Agents** | How do you think a team or person outside your organization could be most helpful to you with implementing PatientToc? |
| **5. Key Stakeholders** | What steps would need to be taken to encourage individuals to commit to using PatientToc? Who would be the key individuals to engage? How does word get out to them? |
| **6. Intervention Participants** | How would you or your colleagues communicate to patients about PatientToc? What do you think patient workflow with PatientToc should look like? |
| **C. Executing** | *[Not applicable]* |
| **D. Reflecting & Evaluating** | If we were to implement it here, what information should be collected to evaluate PatientToc implementation? |

- Anything we haven’t talked about that we need to cover?
- Any closing thoughts or recommendations for us?

**Demographic Data to Collect Verbally Following the Interview:**

**All numbering will start with “Rph” then the number**

1. Age: ___________ years

□ Prefer not to answer_999_

2. Gender

□ Male_1_

□ Female_2_

□ Prefer not to answer_3_

3. Ethnicity

□ Hispanic/Latino_1_

□ Not Hispanic/Latino_0_

□ Prefer not to answer_2_

4. Race

□ White/Caucasion_1_

□ Black/African American_2_

□ Asian_3_

□ American Indian/Alaska Native_4_

□ Native Hawaiian or Other Pacific Islander_5_

□ More than one race_6_

□ Prefer not to answer_7_

5. Type of pharmacy degree received

□ BS_1_

□ PharmD_2_

□ Both BS and PharmD_3_

6. Type of pharmacy residency completed (mark all that apply)?

□ None_1_

□ PGY-1 community_1_

□ PGY-1 managed care_1_

□ PGY-1 emphasis in ambulatory care_1_

□ PGY-2 ambulatory care_1_

□ Other_1______________

7. Other training (fellowship, MS, etc.) completed:____________________

8. Year completed terminal degree and/or initial training (e.g,, residency immediately following graduation completed, began working on own as a pharmacist):____________

9. Certifications or other special training related to medication adherence and/or MTM?______________

10. Year employment began at THIS pharmacy (in any capacity, not necessarily as a pharmacist): _____________

11. Year employment began as a pharmacist at this pharmacy.

12. Current position at this pharmacy:

□ Owner_1_

□ Manager_2_

□ Staff Pharmacist_3_

□ Other_4__________________________

13. Approximate percent of hours per week working directly with patients (including filling prescriptions): ______________%

Interview guide: Pharmacy Staff (i.e., locations where PatientToc NOT implemented)

Study ID#: ________________

**Opening script:**

As a reminder, I’m going to ask you about your ideas related to PatientToc implementation. Please remember that there are no right or wrong answers; we just want your opinion. Your responses will be confidential and only information without your name will be shared. Please don’t state any names on the recording. Do you have any questions before I turn on the recorder and we get started?

To begin, please tell me your initial thoughts about PatientToc now that you have seen and been able to spend a few minutes with a tablet..

- How might you approach implementing PT in your pharmacy?
- What kinds of problems might you expect in trying to implement?
- What about PT or how your pharmacy already operates might help with the implementation?
- At this point, do you see anything that might need to change about PT or your workflow to make this work?

*[Interviewer should then proceed to questions from the below list, organized by CFIR domain/construct, to probe on constructs not addressed or only partially addressed by participant in their summary. It is expected that not all questions will be asked.]*

Examples of questions planned to be loaded into PatientToc for future implementation in community pharmacies. Interviewer should refer to these and tablet to help describe the planned intervention:

[From the BMQ] How much problem or concern are you having in the following areas [circle one]

None A little A lot

a. My medication causes side effects. . . . . . . . . . . . . . . . . . . . . . . . . . 0 1 2

b.It is hard to remember all the doses . . . . . . . . . . . . . . . . . . . . . . . . . 0 1 2

c. It is hard to pay for the medication . . . . . . . . . . . . . . . . . . . . . . . . . 0 1 2

d.It is hard to open the container. . . . . . . . . . . . . . . . . . . . . . . . . . . . . 0 1 2

e. It is hard to get my refill on time . . . . . . . . . . . . . . . . . . . . . . . . . . . 0 1 2

f. It is hard to read the print on the container . . . . . . . . . . . . . . . . . . . 0 1 2

g. The dosage times are inconvenient . . . . . . . . . . . . . . . . . . . . . . . . . 0 1 2

h. My medication causes other problem or concern . . . . . . . . . . . . . . . 0 1 2

If other problem or concern, please explain: ________________________________________________________________

| **CFIR Domain/Construct/Sub-Construct** | **Questions & Probes** |
| --- | --- |
| **I. Intervention Characteristics** |  |
| **A. Intervention Source** | For PatientToc™ to be implemented at your pharmacy, who would need to make the decision? What would you think about the possibility of implementation? |
| **B. Evidence Strength & Quality** | What kind of information or evidence would be needed for you to think PatientToc could work in your pharmacy? |
| **C. Relative Advantage** | How does PatientToc™ compare to other alternatives that may have been considered or used previously? What advantages does it have? Disadvantages? Are there other interventions you believe would be more beneficial? Why is that? |
| **D. Adaptability** | What kind of changes or alterations to PatientToc™ would be needed to make it work in your pharmacy? Why is that? |
| **E. Trialability** | Why types of PatientToc piloting would be needed prior to full-scale implementation at your pharmacy? Why is that? |
| **F. Complexity** | How complicated do you think it would be to use PatientToc™ in your pharmacy? How complicated or not complicated do you think it would be for patients to answer these types of questions [researcher refers to examples from the BMQ, above] in PatientToc when visiting your pharmacy? Why do you say that? What changes, if any, would be needed to make it less complicated? About how much time do you think it would take patients to answer questions like this? How likely or unlikely do you think it would be for patients to have that much time? What would help or not help? How, if it all, do you think the patient “flow” during pharmacy visits might need to change for it to work? What approach do you think would be best for patients taking multiple medications? With multiple medication therapy problems identified? |
| **G. Design Quality & Packaging** | What resources or toolkits, if any, would you want to help implement PatientToc™ in your setting? What other resources, if any, might be ideal? |
| **H. Costs** | What costs would you want to consider and/or measure when deciding to implement PatientToc™ in your setting? |
| **II. Outer Setting** |  |
| **A. Patient Needs & Resources** | How do you think patients would respond to PatientToc? Any barriers to use? Examples? What alterations do you think would be needed made to meet specific needs/preferences? |
| **B. Cosmopolitanism** | What kind of information exchange/discussions would you anticipate having with others outside your setting, related to PatientToc if it were implemented? |
| **C. Peer Pressure** | Can you tell me what you know about any other organizations that have implemented PatientToc or other similar programs? To what extent would implementing PatentToc provide an advantage for your organization compared to other organizations in your area? Examples? |
| **D. External Policies & Incentives** | What kind of local, state, or national performance measures, policies, regulations, or guidelines might influence the decision to implement PatientToc? Why? |
| **III. Inner Setting** |  |
| **A. Structural Characteristics** | How would the infrastructure of your organization (social architecture, age, maturity, size, or physical layout) affect the implementation of PatientToc? How would the infrastructure facilitate/hinder implementation? Examples? What kind of infrastructure changes would be needed? What would this process entail? |
| **B. Networks & Communication** | How do you typically find out about new information, such as new initiatives like PatientToc if it were implemented? |
| **C. Culture** | How do you think your organization's culture (general beliefs, values, assumptions that people embrace) would affect the implementation of PatientToc? To what extent are new ideas embraced and used to make improvements in your organization? Can you describe a recent example? |
| **D. Implementation Climate** | What would you expect the general level of receptivity in your organization to implementing PatientToc? Why? |
| **1. Tension for Change** | Is there a strong need for PatientToc? Why or why not? Do you think others at your organization would agree? Why/why not? |
| **2. Compatibility** | How well would PatientToc fit with existing work processes and practices in your setting? How would PatientToc be integrated into existing processes? |
| **3. Relative Priority** | How would you prioritize getting PatientToc implemented relative to other initiatives that are happening? Why is that? |
| **4. Organizational Incentives & Rewards** | What kinds of incentives would be needed to help ensure that the implementation PatientToc is successful? What would be your personal motivation for ensuring success? |
| **5. Goals & Feedback** | How does PatientToc align with pharmacy goals? |
| **6. Learning Climate** | To what extent do you feel like you can try new things to improve your work processes? Why is that? |
| **A. Readiness for Implementation** | *[Addressed through sub-constructs below]* |
| **1. Leadership Engagement** | What level of endorsement or support for PatientToc would you anticipate from senior leaders at your organization? |
| **2. Available Resources** | What resources would be needed to successfully implement PatientToc? |
| **3. Access to Knowledge & Information** | What kind of training about PatientToc would you need to receive? |
| **IV. Characteristics of Individuals** |  |
| **A. Knowledge & Beliefs about the Intervention** | *[Will evaluate/code based on other responses]* |
| **B. Self-Efficacy** | *[Will evaluate/code based on other responses]* |
| **C. Individual Stage of Change** | How prepared do you feel to use PatientToc? Why? |
| **D. Individual Identification with Organization** | *[Will evaluate/code based on other responses]* |
| **E. Other Personal Attributes** | *[Will evaluate/code based on other responses]* |
| **V. Process** |  |
| **A. Planning** | What plan would you suggest for implementing PatientToc? |
| **B. Engaging** | *[Assessed by sub-constructs below]* |
| **1. Opinion Leaders** | Who would be the key influential individuals to get on board with PatientToc? |
| **2. Formally Appointed Internal Implementation Leaders** | Who would you anticipate leading implementation of PatientToc? *[Just describe their role; not an actual name]* How would you anticipate this person coming into this role? Appointed? Volunteered? Voluntold? How might implementation go differently if another individual led it? Who else do you think would be involved with leading the implementation? |
| **3. Champions** | Other than the formal implementation leader, are there people in your organization who you think would champion (go above and beyond what might be expected) PatientToc? |
| **4. External Change Agents** | How do you think a team or person outside your organization could be most helpful to you with implementing PatientToc? |
| **5. Key Stakeholders** | What steps would need to be taken to encourage individuals to commit to using PatientToc? Who would be the key individuals to engage? How does word get out to them? |
| **6. Intervention Participants** | How would you or your colleagues communicate to patients about PatientToc? What do you think patient workflow with PatientToc should look like? |
| **C. Executing** | *[Not applicable]* |
| **D. Reflecting & Evaluating** | If we were to implement it here, what information should be collected to evaluate PatientToc implementation? |

- Anything we haven’t talked about that we need to cover?
- Any closing thoughts or recommendations for us?

**Demographic Data to Collect Verbally Following the Interview:**

**All numbering will start with “STAFF” then the number**

1. Age: ___________ years

□ Prefer not to answer_999_

2. Gender

□ Male_1_

□ Female_2_

□ Prefer not to answer_3_

3. Ethnicity

□ Hispanic/Latino_1_

□ Not Hispanic/Latino_0_

□ Prefer not to answer_2_

4. Race

□ White/Caucasion_1_

□ Black/African American_2_

□ Asian_3_

□ American Indian/Alaska Native_4_

□ Native Hawaiian or Other Pacific Islander_5_

□ More than one race_6_

□ Prefer not to answer_7_

5. Position within pharmacy

□ Pharmacy Technician_1_

□ Other (Briefly summarize role and how unique from a pharmacy technician)_2___________________

6. Highest education competed

□ High School/GED_1_

□ Some college_2_

□ Associate’s degree_3_

□ Bachelor’s degree_4_

□ Other_5______________

7. Summary of pharmacy training/certification completed (include a description of training or certifications taken to become a staff member in the pharmacy)_______________

8. Summary of training/ certification related to MTM or medication adherence (such as med sync):_____________________

9. Year completed terminal degree and/or training to be a pharmacy staff member:____________

10. Year employment began at THIS pharmacy: _____________

11. Year employment began as a pharmacy staff member:_____________________

12. Approximate percent of hours **per** week spent working directly with patients (including working with pharmacists to fill prescriptions): ______________%

Interview guide: Pharmacy Patients (i.e., locations where PatientToc NOT implemented)

Study ID#: ________________

***[Note: Below are draft script/interview guide/demographic items for the semi-structured interviews. These will be piloted with a small number of patients and minor modifications and/or deletions/additions may be made prior to data collection to ensure items are clear, as relevant as possible, and comprehensive.]***

**Opening script:**

As a reminder, I’m going to ask you about your experiences and/or ideas related to PatientToc implementation. Please remember that there are no right or wrong answers; we just want your opinion. Your responses will be kept confidential and only information where your name has been removed will be shared. Please don’t state any names on the recording. Do you have any questions before I turn on the recorder and we get started?

To begin, please tell me your initial thoughts about PatientToc now that you have seen and been able to spend a few minutes with a tablet. *[Interviewer should then proceed to questions from the below list, organized by CFIR domain/construct, to probe on constructs not addressed or only partially addressed by participant in their summary. It is expected that not all questions will be asked.]*

*[Examples of questions planned to be loaded into PatientToc for future implementation in community pharmacies. Interviewer should refer to these and tablet to help describe the planned intervention*]:

3. [From the BMQ] How much problem or concern are you having in the following areas [circle one]

None A little A lot

a. My medication causes side effects. . . . . . . . . . . . . . . . . . . . . . . . . . 0 1 2

b.It is hard to remember all the doses . . . . . . . . . . . . . . . . . . . . . . . . . 0 1 2

c. It is hard to pay for the medication . . . . . . . . . . . . . . . . . . . . . . . . . 0 1 2

d.It is hard to open the container. . . . . . . . . . . . . . . . . . . . . . . . . . . . . 0 1 2

e. It is hard to get my refill on time . . . . . . . . . . . . . . . . . . . . . . . . . . . 0 1 2

f. It is hard to read the print on the container . . . . . . . . . . . . . . . . . . . 0 1 2

g. The dosage times are inconvenient . . . . . . . . . . . . . . . . . . . . . . . . . 0 1 2

h. My medication causes other problem or concern . . . . . . . . . . . . . . . 0 1 2

If other problem or concern, please explain: ________________________________________________________________

| **CFIR Domain/Construct/Sub-Construct** | **Questions & Probes** |
| --- | --- |
| **B. Evidence Strength & Quality** | What kind of information or evidence would you like to see to have the sense that PatientToc would help your pharmacy to better serve you? |
| **C. Relative Advantage** | How does PatientToc™ compare to other similar technologies that you may have used previously? What advantages does it have? Disadvantages? Are there other interventions you believe would be more beneficial to patients? Why is that? |
| **F. Complexity** | How hard do you think it would be to use PatientToc™? How easy does it seem to you? How hard would it be to answer these types of questions [researcher refers to examples from the BMQ, above] in PatientToc when visiting your pharmacy? Why do you say that? What changes, if any, could make it easier? About how much time do you think it would take to answer questions like this? |
| **G. Design Quality and Packaging** | What design features of PatientToc do you think would help or hinder its use? |
| **A. Patient Needs & Resources** | When you pick up medications here, what types of questions or concerns do you most often have about your medications? (Interviewer asks open ended before giving a list) For example, cost, insurance, side effects, benefits, is the medicine really needed still, interactions, which ones you can take together, other issues? How aware or unaware of your questions/concerns/preferences are your pharmacists and staff? Normally, how much time would you have available to fill out something like PatientToc when you come to pick up your medicines? Normally, how much time would you have available to talk with the pharmacist about your medication after you filled out something like PatientToc? How feasible or not feasible would it be to come here early to fill out something like PatientToc? About how many minutes early would be feasible? How many of your medicines would you want to discuss with a pharmacist each visit after filling out something like PatientToc? How would you choose which medicine(s) to focus on? What alterations do you think would be needed so PatientToc meets your specific needs/preferences? |
| **A. Knowledge & Beliefs about the Intervention** | Now that you have seen PatientToc, what do you think of it? How helpful do you think it would be to you? What do you think should be changed about how it works? |
| **B. Self-Efficacy** | How confident or not confident would you feel using PatientToc? Why? How confident are you that it could help you and the pharmacist talk about your top concerns related to your medications? |
| **6. Intervention Participants** | How does your pharmacy typically communicate with you? How would you expect your pharmacy to communicate with you about PatientToc if it were implemented?  At what point during your pharmacy visit would it make those sense here for patients to use PatientToc (e.g., first, complete questions on tablet and then, approach pharmacy counter)? How, if at all, do you think the process/steps you take when visiting the pharmacy (e.g., dropping off prescription, waiting for prescription, talking to the pharmacist, etc.) might need to change for it to work best for you? |
| **D. Reflecting & Evaluating** | If we were to try PatientToc out here, what information should we collect from patients to determine how well it works |

**Wrap up questions:**

-Anything we haven’t talked about that we need to cover?

-Any closing thought or recommendations for us?

**Demographic Data to Collect Verbally Following the Interview:**

1. Age: ___________ years

□ Prefer not to answer_999_

2. Gender

□ Male_1_

□ Female_2_

□ Prefer not to answer_3_

3. Ethnicity

□ Hispanic/Latino_1_

□ Not Hispanic/Latino_0_

□ Prefer not to answer_2_

4. Race

□ White/Caucasion_1_

□ Black/African American_2_

□ Asian_3_

□ American Indian/Alaska Native_4_

□ Native Hawaiian or Other Pacific Islander_5_

□ More than one race_6_

□ Prefer not to answer_7_

5. About how often to do you visit THIS pharmacy (if response is not straightforward then use “other” and include nuances (such as caregiver picks up prescriptions)?

□ At least once per week_1_

□ Less than every week but more than once per month_2_

□ About once per month_3_

□ About once every three months_4_

□ Other_5____________

6. How many different chronic conditions do you have that require routine prescription medication use? You do not need to specify which conditions___________

If you aren't sure what a chronic condition is, here are some examples:

Hypertension (High blood pressure)

Hyperlipidemia (High cholesterol)

Diabetes

Heart Failure

Atrial Fibrillation

Arthritis (Osteoarthritis and Rheumatoid)

Hepatitis (Chronic Viral B & C)

Cirrhosis or other liver disease

Depression

Asthma

HIV/AIDS

Autism Spectrum Disorders or ADD/ADHD

Narcolepsy or other sleep/wake disorder

Cancer (Breast, Lung, and Prostate)

Chronic pain

Irritable Bowel Syndrome

Inflammatory Bowel Disease (Crohn's Disease or

ulcerative colitis)

Lupus or other autoimmune disease

Thyroid disorder

Epilepsy or other seizure disorder

Hypothyroidism or hyperthyroidism

Alzheimer's Disease and related Dementia

Substance-use disorder

Psoriasis or other chronic skin condition

7. How many different medications do you use regularly (count each drug name once- don’t double count if taking multiple doses/ forms of same drug)?

Prescription___________

Non-prescription/OTC___________

Herbals/supplements____________

8. Is English the subject’s primary language? Yes/No (investigator to answer)

9. Was a translator used during this interview? Yes/No (investigator to answer)
