# Supplementary material for: Preparing for the spread of patient-reported outcome (PRO) data collection from primary care to community pharmacy: a mixed-methods study
Source: Implement Sci Commun. 2022 Mar 14;3:29. doi: 10.1186/s43058-022-00277-3 (PMC8919161; doi:10.1186/s43058-022-00277-3)
Supplement: Supplementary file 3 — Additional file 3. Codebook. [file 43058_2022_277_MOESM3_ESM.docx]

**CFIR Codebook**

**Considerations:** [www.cfirguide.org](http://www.cfirguide.org) was consulted to gain a deeper understanding of CFIR definitions. Of note, there are 39 CFIR constructs (CC) listed here <https://cfirguide.org/constructs/> when exported to Excel from the site. However, the CFIR Codebook Template (<https://cfirguide.org/tools/tools-and-templates/>) guided codebook development and has 41 constructs when exported to Word from the website. Major and minor differences are detailed below.

Major Differences

1. Key Stakeholders (our code “5B5_KeyStakeholders”) and Innovation Participants (our code 5B6_InterventionParticipants) are included in the codebook template (temp) under *Domain 5 Process* but NOT in the listed CFIR constructs (CC)
   1. We included these two codes in our Level 1” (see definition below) codes totaling 41 codes (temp).

Minor Differences

1. **Domain 1: Intervention Characteristics** (CC) vs. Innovation Characteristics (temp)
   1. We used Intervention Characteristics (CC)
2. **Construct: Intervention source** (CC) vs. Innovation Source (temp)
   1. We used Intervention Source (CC) our code 1A_InterventionSource
3. **Construct: Knowledge & Beliefs about the Intervention** (CC) vs. Knowledge & Beliefs about the Innovation (temp)
   1. We used Knowledge & Beliefs about the Intervention (CC) our code 4A_KnowBeliefsInterven

The CFIR Codebook Template only included CFIR definitions and coding criteria; we include other information including our deductive and inductive sub-codes and definitions, notes, and audit trail.

Our study “data dictionary” and “interview guides” were consulted to identify applicable stakeholders and to frame defintions for each CIFIR construct; the study “Research Strategy” was consulted to adapt wording for definitions.

“Stakeholders” – operationalized as any of our human subjects

“Perceptions” – operationalized as both explicitly stated as well as implied through actions, body language etc. through interviews, contextual inquiries, and/or observations

Note: many construct definitions are written as past tense but for pharmacies should be interpreted in future tense.

# **Deductive Level 1 and 2 Coding**

- Level 1 (broad codes) = used to broadly group data into the 41 CFIR constructs
  - **41 “Level 1” broad codes**
- Level 2 (sub-codes) = potential barriers (B) to PatientToc implementation (by CFIR construct) in community pharmacies, potential facilitators (F) to PatientToc implementation (by CFIR construct) in community pharmacies, and actionable recommendations (R) for PatientToc implementation (by CFIR construct) in community pharmacies to further delineate each level 1 broad code
  - 41 level 1 broad codes X 3 (B, F, R) = **123 “Level 2” sub-codes**

# **Inductive Level 3 Coding**

- Level 3 (sub-codes) = inductive codes created in response to actual data coded for B, F, R to further delineate each level 2 sub-code
  - **234 “Level 3” sub-codes**

**General Coding Rules:**

- When two codes are in question for a passage, consider the primary meaning of the passage to assign code; consider what the participant is truly saying. Analysts should wish to err on the side of inclusion. NO double coding is permitted.

**Table 1. Aim 1 Code Book: Deductive Level 1 (broad codes), 2 (sub-codes) and Inductive Level 3 (sub-codes)**

| **Level 1 broad codes (CFIR constructs)** | **Level 2 sub-codes** | **Definition/Exclusion/Inclusion criteria** | **Level 3 sub-codes (inductive)** | **Definition of Level 3 codes** |
| --- | --- | --- | --- | --- |
| **1. Intervention Characteristics** |  |  |  |  |
| 1A_InterventionSource | Intervention Source_B  Intervention Source_F  Intervention Source_R | Definition: Perception of key stakeholders (explicitly noted in guides) about whether PatientToc is externally or internally initiated and/or implemented.  Inclusion Criteria: Include statements about the origin of PatientToc and the extent to which key stakeholders view PatientToc as internal to the organization e.g., an internally developed program, or external to the organization, e.g., a program coming from the outside (research project, state or federal policy etc.).  Code statements about key personnel involved in implementing PatientToc or statements related to who participated in the decision process to implement PatientToc to [Engaging](http://cfirwiki.net/wiki/index.php?title=Engaging), as an indication of early (or late) engagement. | 1. Singl_decis_maker_B 2. Extern_Org_Factors_B 3. Intern_Org_factors_B 4. Singl_decis_maker_R 5. Consensus_R 6. Intern_Org_factors_R 7. Extern_Org_factors_R | 1B. Challenges related to single individual internal to the pharmacy location being difficult to convince  2B. Shared space with players external to the pharmacy such as non-affiliated medical office leading to potential complications from having many players from different organizations involved.  3B. Ways in which internal pharmacy organization factors , such as the number of people to go through to get approval, impact the decision making.  1R. One person should make decision to implement PT  2R. Solicit input on decision to implement PT from patients also include CFO, marketing, all levels of pharmacy management and leadership. Pharmacies that typically use a shared consensus approach to decision making would be helpful in implementing PT. Specifically, code statements about soliciting input and how it generates buy in needed such as from staff, pharmacists, i.e., co-op style, as well departments impacted such as marketing and finance here.  3R. It is expected that having a formal organizational process in place to gain approval from applicable individuals and systems will be helpful to implementing PT. Specifically, for pharmacies that have more than one site to consider.  4R. Consider whole system such as entities “external” to the pharmacy e.g., hospital and clinics, but are located in one physical site. |
| 1B_EvidStrengthQual | EvidStrengthQual_B  EvidStrengthQual_F  EvidStrengthQual_R | Definition: Stakeholders’ perceptions of the quality of evidence supporting the belief that implementation of PatientToc will “do what its supposed to do.” This will depend on what PatientToc is supposed to do in the given setting. For example using it to improve patient care in a primary care setting, to collect and monitor CAHPS scores, to identify and act on medication non-adherence.  Inclusion Criteria: Include statements regarding awareness, strength, and quality of evidence, supporting or not supporting PatientToc. Also includes statements about desire for different types of evidence (e.g., research study, data from a peer pharmacy, evidence from the literature.)  Code statements regarding the receipt of evidence as an engagement strategy to [Engaging](http://cfirwiki.net/wiki/index.php?title=Engaging): Key Stakeholders.  Code descriptions of use of results from local or regional pilots to [Trialability](http://cfirwiki.net/wiki/index.php?title=Trialability). | 1. Not_evidence-based_B 2. Neg_customer _serv_B 3. No_ROI_B 4. Good_ROI_F 5. Participation_rate_F 6. Clinical_pt_outcomes_F 7. Good_ROI_R 8. Participation_rate_R 9. Clinical_pt_outcomes_R 10. Workflow_R 11. Gen_optimism_R | 1B. There is a belief that PatientToc is not evidence-based medicine but “a tool.” General statements about needing to be convinced it is worth it are coded here.  2B. Staff not being able to attend to patient needs (satisfaction, frustration level) due to time needed for PatientToc  3B. Whether PT is perceived as worth the time and whether it is doing what it’s supposed to do. Some benefits can be difficult to measure.  1F. There is a belief that using PatientToc is worth patient and pharmacy time because it is expected to result in improvement in scores and metrics including ability to report this out.  2F. There is a belief that PT will be utilized by pharmacy staff and patients.  3F. There is a belief that PT will be able to assist with things clinic is already looking at, adherence, identify drug therapy problems, increased interventions, extent to which disease states are being managed, and/or improved patient satisfaction.  1R. Improvement in scores and metrics including ability to report this out.  2R. Patients and pharmacist, show that you are gathering info from patients  3R. Outcomes related to clinical indicators such as blood pressure and labs including safety such as hospitalization rates. Perceived as beneficial to patient, patient acceptance level, and patient satisfaction, pharmacy’s ability to communicate with patients, Capture what interventions are being made by pharmacists because of PatienTOC.  4R. Consider impact to workflow including from system integration.  5R. Interviewee feels that PatientToc appears to be a good idea for the pharmacy. |
| 1C_ RelAdvantage | RelAdvantage_B  RelAdvantage_F  RelAdvantage_R | Definition: Stakeholders’ perception of the advantage of implementing PatientToc as a tool to collect and respond to PROs related to medication adherence versus other alternatives for collecting and using PROs and/or improving medication adherence.  Inclusion Criteria: Include statements that demonstrate PatientToc is better (or worse) than existing alternatives.  Code statements that demonstrate a strong need for PatientToc and/or that the current situation is untenable and code to [Tension for Change](http://cfirwiki.net/wiki/index.php?title=Tension_for_Change). | 1. Prefer paper_B   1. Feedback_tool_F   1. Easier_alt_F 2. Privacy_F 3. Pt_centered_R 4. Increase_efficiency_R | 1B. Prefers using paper over technology. Stakeholders would prefer not to use PatientToc (at all or prefer a different system) due to various reasons such as: a. site doesn’t care about collecting the information, or would not use this type (survey) of approach due to site’s desires and/or culture, OR redundancy with other systems e.g., double capturing patient data, whether asking the same question in PatientToc or being used in conjunction with another system.  1F. The expectation that PatientToc will provide a helpful way to get patient feedback  2F. The expectation that PatientToc is an easier, more efficient, or simpler alternative to the current system.  3F. The expectation that patients may feel they have more privacy with PatientToc.  1R. Use PatientToc to help create an atmosphere of patient centered care. Especially, if it can be used to help give a “personal touch”.  2R. Use PatientToc to increase the efficiency of workflow and more focused time spent with patients compared to the current system. |
| 1D_ Adaptability | Adaptability_B  Adaptability_F  Adaptability_R | Definition: The degree to which PatientToc can be adapted, tailored, refined, or reinvented to meet the needs of a variety of clinics and community pharmacies.  Inclusion Criteria: Include statements regarding the (in)ability to adapt PatientToc to various community pharmacies and clinics.  Suggestions for improvement can be captured in this code but should not be included in the rating process, unless it is clear that the participant feels the change is needed but that the program cannot be adapted. However, it may be possible to infer that a large number of suggestions for improvement demonstrates lack of compatibility, see exclusion criteria below.  Code statements in which PatientToc did or did not need to be adapted to [Compatibility](http://cfirwiki.net/wiki/index.php?title=Compatibility). | 1. Questionnaire_B 2. Privacy_B 3. Interface_systems_B 4. Language_B 5. Questionnaire_F 6. Privacy_F 7. Interface_systems_F 8. Process_F 9. Language_F 10. Questionnaire _R 11. Pt edu_R 12. Interface_Systems_R 13. Pt_communicate_R 14. Language_R | 1B. Lack of ability to modify questionnaires or length of surveys  2B. Privacy (lack of privacy) that PatientToc provides – including comments about mobility or lack of a private space  3B. Lack of ability to interface w EHR and/or pharmacy systems  4B. Lack of ability to adapt to people of different languages  1F. Perceived ability to modify questionnaires/surveys is a facilitator to use PatientToc  2F. Perceived ability to keep Patient information within PT safe/private helps PatientToc implementation by making patients feel safer  3F. Perceived ability to interface with EHR and/or pharmacy systems helps with PatientToc implementation  4F. Perceived ability to change where/when PatientToc is administered to patients – different places in the process – allows PatientToc to be more easily implemented to different sites  4F. Perceived ability to adapt to people who speak languages other than English would be helpful in helping patients overcome the language barrier.  1R. Ensure stakeholders have the ability to modify questionnaires/surveys for use in different services(“use cases”) beyond typical dispensing and counseling, such as MTM, MedSync, etc.  2R. Ensure PT is able to give patients educational material  3R. Ensure PatientToc is able to interface w the EHR and/or pharmacy systems in place.  4R. Ensure PT has the ability to be used as a communication tool between the pharmacy and the patient. An example of this would having the patient be able to request a refill or have PT in an “app” format.  5R. Ensure PT is able to adapt to people who speak a language other than English |
| 1E_Trialability | Trialability_B  Trialability_F  Trialability_R | Definition: The ability to test PatientToc on a small scale in the clinic/pharmacy, and to be able to reverse course (undo implementation of PatientToc) if warranted.  Inclusion Criteria: Include statements related to whether the site piloted PatientToc in the past or has plans/interest to in the future, and comments about whether they believe it is (im)possible to conduct a pilot.  Code descriptions of use of results from local or regional pilots to [Evidence Strength & Quality](http://cfirwiki.net/wiki/index.php?title=Evidence_Strength_%26_Quality). | 1. Phased trials_F 2. Trial_benefit_F 3. Site_contact_R 4. Phased trials_R 5. Conduct_trial_R | 1F. The pharmacy or clinic already has a phased trial approach in place for conducting a pilot. For example, starting small with different phases of focused trials such as 5 per day, limited to certain staff members, one clinic, Specified group or type of patient such as those waiting for prescription fills. Take time needed to carry out a trial such as 6-month trial.  2F. Includes reasons why a pilot is perceived as an advantageous step in implementation including general agreement.  1R. Appoint one person who will have and hold accountability.  2R. Have different phases to rollout such as easing patients into it, beginning with a common goal, and beginning with staff members. Target those who would be receptive and gain benefit, in a program already, or with new Rxs. Trials cross different people or patient types and different situations.  3R. Use a trial period to elicit patient feedback and tests different kinds of questions. |
| 1F_Complexity | Complexity_B  Complexity_F  Complexity_R | Definition: Perceived difficulty of using PatientToc, reflected by duration, scope, radicalness, disruptiveness, centrality, and intricacy and number of steps required to implement PatientToc.  Inclusion Criteria: Code statements regarding the complexity of using PatientToc to collect and respond to PROs related to medication adherence.  Code statements regarding the complexity of implementation to the appropriate CFIR code, e.g., difficulties related to space are coded to Available Resources and difficulties related to engaging participants in a new program are coded to [Engaging](http://cfirwiki.net/wiki/index.php?title=Engaging): Innovation Participants. | 1. PT_Time_B 2. User_friendliness_B 3. Lim_tech_exper_B 4. PT_Time_F 5. User_friendliness_F 6. PT_Time_R 7. User friendliness_R | 1B. Amount of time to do PatientToc is too long  2B. PatientToc is perceived as overly complex or difficult to use  3B. PatientToc is difficult to use due to unfamiliarity with technology. Includes statements relating to the elderly not being able to use the technology due to not understanding. These are barriers to implementation.  1F. Length of time to use PatientToc is quick for patients  2F. PatientToc is easy to use for patients  1R. Recommendation of how long patients would be willing to spend using PatientToc  2R. Recommendations to make PatientToc more or less complex to use for participants |
| 1G_DesignQualPack | DesignQualPack_B  DesignQualPack_F  DesignQualPack_R | Definition: Perceived excellence in how PatientToc is bundled, presented, and assembled.  Inclusion Criteria: Include statements regarding the quality of the materials and packaging provided by PatientToc.  Code statements regarding the presence or absence of materials to [Available Resources](http://cfirwiki.net/wiki/index.php?title=Available_Resources).  Code statements regarding the receipt of materials as an engagement strategy to [Engaging](http://cfirwiki.net/wiki/index.php?title=Engaging). | 1. VTP_B 2. Tech_glitch_B 3. VTP_F 4. VTP_R | 1B. VTP = voice, text, and packaging. Several barriers related to VTP of PatientToc exist such as uses an improper or non-inclusive dialect of a language. Voice feature is not loud enough, font size is too small, tablet is not durable enough.  2B. PatientToc experiences technical glitches, freezing, works too slowly, and similar issues.  1F. VTP = voice, text, and packaging. Several facilitators related to VTP features of PT exist. These include having the voice and the ability to mute it is a helpful feature as well as the ability to use earbuds with the voice feature. User friendly formatting and interface of the tablet, helpful color scheme, and appropriate size of font and tablet itself.  1R. VTP = voice, text, and packaging. Improve VTP of PatientToc, such as creating a PatientToc app for phones, more voice options for the PatientToc tablet, incorporate more languages into the tablet, have chargers readily available, and use a case for the tablet. Additional improvements include: increase font size, have different color schemes available for color blind patients, improve the ease of use by including free response type boxes, having clearer prompts for use of tablet, etc. |
| 1H_Cost | Cost_B  Cost_F  Cost_R | Definition: Costs of using PatientToc and costs associated with implementing PatientToc including investment, supply, and opportunity costs.  Inclusion Criteria: Include statements related to the cost of PatientToc and its implementation.  Code statements related to physical space and time to [Available Resources](http://cfirwiki.net/wiki/index.php?title=Available_Resources). In a research study, exclude statements related to costs of conducting the research components (e.g., funding for research staff, participant incentives). | 1. Cost justification_B 2. Hardware needs_B 3. Tech_software_B 4. Good ROI_F 5. Hardware needs_F 6. Tech_software_F 7. Tech_software_R | 1B. The justification of cost including to higher leadership is expected to be or may be challenging.  2B. Physical tablet needs including number of tablets, replacement, theft, physical product maintenance, and accessories are perceived to generate additional costs that could be a barrier to implementation. Pros and cons of portability mobile versus stationary tablets and relationship to cost.  3B. Cost to run program start-up and maintenance; Cost to integrate dispensing system, EMR, delivery, etc. are perceived as a potential a barrier to implementation.  1F. There is an expectation that while costs will be accrued in staff time, the pharmacy will make money or achieve improved other goals of business, such as patient satisfaction.  2F. Cost of some accessories are inexpensive such as earbuds. The ability to have tablets be mobile in use allows them to be in greater number of patient areas so fewer tablets.  3F. Cost to run program start-up and maintenance is expected to be affordable.  1R. Recommendations for program maintenance to be a yearly flat fee vs per script or short-term vs. long-term commitment. |
| **2. Outer Setting** |  |  |  |  |
| 2A_ PtNeedsResources | PtNeedsResources_B  PtNeedsResources_F  PtNeedsResources_R | Definition: The extent to which the needs of patients served by the organization, as well as barriers and facilitators to meet those needs, are accurately known and prioritized by the organization.  Inclusion Criteria: Include statements demonstrating/not demonstrating awareness of the needs and resources of patients served by the organization. Analysts may be able to infer the level of awareness based on statements about: 1. Perceived need for PatientToc based on the needs of patients served by the organization and if PatientToc will meet those needs; 2. Barriers and facilitators to patient use of PatientToc; 3. Patient feedback on PatientToc such as dis/satisfaction. In addition, include statements that capture whether or not awareness of the needs and resources of patients influenced the implementation or adaptation of PatientToc.  Code statements that demonstrate a strong need for PatientToc and/or that the current situation is untenable to [Tension for Change](http://cfirwiki.net/wiki/index.php?title=Tension_for_Change).  Code statements related to engagement strategies and outcomes, e.g., how PatientToc participants became engaged with PatientToc to [Engaging](http://cfirwiki.net/wiki/index.php?title=Engaging): PatientToc Participants. | 1. Skepticism_B 2. Phrm_Time_B 3. Communication_B 4. SDOH_B 5. Tech_concerns_B 6. Patient experience_F 7. Phrm_Time_F 8. Communication_F 9. SDOH_F 10. Med_Rel_Needs_F 11. Patient experience_R 12. Phrm_time_R 13. Communication_R 14. SDOH_R 15. Med_Related_Needs_R | 1B. Statements including fears of malintent or unintended consequences or need in general for PatientToc such as data being collected and misused by outside entities such as insurance companies.  2B. Code statements regarding patients not wanting or able to spend time at the pharmacy or staff’s perceptions that patients don’t have time to spend at the pharmacy here. Code statements about challenges specific to length of time participants spend on PatientToc to PT_Time_B (complexity).  3B. Code statements about patients not wanting to share information with pharmacy that is personal related to embarrassment, shame, stigmatized medical conditions like depression, and financial concerns.  4B. Social determinants of health (SDOH) (<https://www.healthypeople.gov/2020/topics-objectives/topic/social-determinants-of-health>) related to specific populations or individual preferences. Considerations of issues related to patients who do not physically come to pharmacy (i.e. due to limited transportation or mobility). Any reasons that result in a patient not understanding such as perceived or real impact of literacy level on ability to interact with device, or not understanding rationale.  5B. Code statements about patients having low comfort levels with or desire for using technology in general, including privacy concerns with using technology specifically.  1F. Use of PatientToc is expected to help the pharmacy/pharmacist to better understand patient experience or satisfaction. Ways that PatientToc could personalize the interaction at pharmacy, helps patients feel they and their medicines are known, or improves personal contact are coded here.  2F. The belief PT may improve use of patients’ time. General respect of people’s time, the amount of time they might want to spend in the pharmacy.  3F. The belief that using PT may be more conducive way to share personal information that patients may not otherwise want to share, such as STD or pregnancy information. Belief that PT will accommodate language, vision, and hearing needs.  4F. The belief that PT addresses patient SDOH related needs such as access i.e. delivery, housing, food insecurity or probe into issues they may not know they have. Belief that PT will accommodate specific patient populations such as group homes, discharge patients, younger generation, other SDOH.  5F. Belief that PT can be tailored to specific individual medication-related needs such as pregnancy-related issues, inventory/availability to save trips, larger print, side effects and cost.  1R. Use PatientToc to better understand patient experience or satisfaction. (i.e., to address privacy and physical comfort concerns of patients.)  2R. General respect of people’s time, the amount of time they might want to spend in the pharmacy, and ways PatientToc may improve use of time.  3R. Recommendations regarding how information from PatientToc should be acted on and communicated to patients, as well as how patient should be able to communicate with pharmacy. Code statements about what care teams have access to information. Accommodations should be made for language, vision, hearing needs.  4R. Address patient SDOH related needs such as access i.e. delivery, housing, food insecurity or probe into issues they may not know they have. Need ways to access patients other than physically in pharmacy. Accommodate low literacy and cognitive abilities as well as willingness to participate by talking to people to explain the program/walking through the questions and visual imagery. Includes recommendations for patient populations such as group homes, discharge patients, younger generation, other SDOH.  5R. Tailor to specific individual medication-related needs such as pregnancy-related issues, inventory/availability to save trips, larger print, side effects and cost. |
| 2B_ Cosmopolitanism | Cosmopolitanism_B  Cosmopolitanism_F  Cosmopolitanism_R | Definition: The degree to which an organization is networked with other external organizations.  Inclusion Criteria: Include descriptions of outside group memberships and networking done outside the organization.  Code statements about general networking, communication, and relationships in the organization, such as descriptions of meetings, email groups, or other methods of keeping people connected and informed, and statements related to team formation, quality, and functioning to [Networks & Communications](http://cfirwiki.net/wiki/index.php?title=Networks_%26_Communications). | 1. Integr_B 2. System_F 3. PCP_relat_F 4. Buy_group_F 5. Commun_F 6. Integr_R | 1B. Perceived lack of integration/communication between software of pharmacies, clinics, hospitals, etc.  1F. Pharmacy or clinic is part of a larger health system (operationalized as external to pharmacy) with associated communications as part of this system, which is expected to positively influence PT implementation.  2F. Pharmacy or clinic has good relationships with their associated PCPs (operationalized as external to pharmacy), which is expected to positively influence PT implementation.  3F. Pharmacy is connected to others because it is part of a buying group (operationalized as external to pharmacy), which is expected to positively influence PT implementation.  4F. Pharmacy is connected to a nearby university and/or takes students, has connections with pharmacy societies, and/or connections with local police and fire departments, which is expected to positively influence PT implementation.  1R. Integration of software systems (external to pharmacy) with pharmacy internal software system and PatientToc would provide better patient care. |
| 2C_PeerPressure | PeerPressure_B  PeerPressure_F  PeerPressure_R | Definition: Mimetic or competitive pressure to implement PatientToc.  Inclusion Criteria: Include statements about perceived pressure or motivation from other entities or organizations in the local geographic area or system to implement PatientToc. | 1. Pharm_culture_B 2. Unutilized_B 3. Compet_advan_F 4. Propel_pharm_F | 1B. Implementing PatientToc is not expected by stakeholders to provide a competitive advantage over other pharmacies due to the culture and desire of patients. This is a lack of peer pressure and therefore less incentive to implement PatientToc.  2B. Other competitors are not utilizing a similar software and therefore this would not provide advantage  1F. Implementing PatientToc is expected to position the pharmacy to provide better patient care than competing pharmacies without similar technology. This would give the PatientToc pharmacy a “leg up”.  2F. PatientToc is perceived as a tool to propel the world of pharmacy which is good because there is pressure in the current marketplace to distinguish yourself from other pharmacies |
| 2D_ ExtPolicyIncentives | ExtPolicyIncentives_B  ExtPolicyIncentives_F  ExtPolicyIncentives_R | Definition: External influences which incentivize/provide the impetus for the implementation of PatientToc, including policy and regulations (governmental or other central entity, e.g., Medicare Star Ratings), external mandates, recommendations and guidelines, pay-for-performance, collaboratives, and public or benchmark reporting.  Inclusion Criteria: Include descriptions of external performance measures | 1. STAR ratings_B 2. STAR ratings_F 3. Desire for data/data collection_F | 1B. General negative comments about STAR ratings (i.e., the ratings exist and are known to stakeholders but are not valued by the pharmacy/stakeholders; therefore, the presence of ratings doesn’t create an incentive to PT use)  1F. PatientToc is perceived as having potential to help with STAR ratings in the pharmacy setting  2F. PatientToc is perceived as having potential to help the pharmacy collect data to meet different metrics/regulations(other than STAR). Includes comments perceiving PT as having potential to help the pharmacy collect data on how staff is doing to meet externally mandated performance measures. Also includes statements about “doing what they need to do anyway” with regards to different measures. Also includes statements about the ability of PT implementation being able to affect the site’s bottom line (ex. DIR fees) |
| **3. Inner Setting** |  |  |  |  |
| 3A_StructChacter | StructChacter_B  StructChacter_F  StructChacter_R | Definition: The social architecture, age, maturity, physical layout, and size of an organization.    Inclusion Criteria:  Details regarding age, maturity, size, physical layout, etc., of the organization as it pertains to the possible barriers, facilitators, and recommendations. | 1. New_leadership_B 2. Space_limits_B 3. Lack_pat_contact_B 4. Space_issues_F 5. Imp_relations_F 6. Org_size_F 7. Pat_comfort_F 8. Tablet_access_R   2. Pat_comfort-access_R | 1B. The pharmacy’s recent changes in reporting structure is perceived as likely to create unforeseen barriers to PT implementation with new ownership. Includes any barriers related to being new.  2B. Stakeholders perceive that the pharmacy physical space will create challenges for PatientToc implementation: limited space will cause a lack of patient privacy and confidentiality; There is a perceived Inability to move tablet due a structured waiting area; and currently space dedicated to workflow operations are not optimized or have physical limitations.  3B. The pharmacy provides many services without face to face contact with patients such as with telephone and delivery services and it is perceived that this could be a barrier to using PatientToc.  1F. The ways in which the physical space is or can be organized is perceived as able to accommodate PT, including privacy capabilities and limitations. This includes the need/ability to move the Android tablet.  2F. The relationships the pharmacy has developed with key groups such as with clinics, clientele, and the community have created a feeling of trust with the community which is expected to support PT implementation.  3F. There are aspects of the organization such that the stakeholders believe it could accommodate PT such as the size, age, and co-existence with clinics as part of a larger health care system; specific examples of ways in which it could do so are coded here.  4F. The pharmacy is perceived to have pre-existing strengths which are expected to support the patients’ physical comfortable when using the tablet.  1R. Consider ensuring the ability to move around with the tablet freely or not and balance with concerns such as theft. This includes statements of recommendations for places where the Android tablet should be located, such as with the pharmacy staff behind in the counter or in kiosks.  2R. Consider patient physical comfort and access such as privacy, needing chairs, ability to concentrate, access for individuals with disabilities, and ways in which tablets will be made visible to patients. |
| 3B_NetworkCommun | NetworkCommun_B  NetworkCommun_F  NetworkCommun_R | Definition: The nature and quality of social networks and formal/informal communication within an organization.  Inclusion Criteria: Include statements about general networking, communication, and relationships in the organization, such as descriptions of meetings, email groups, or other methods of keeping people connected and informed, and statements related to team formation, quality, and functioning.  Code statements related to implementation leaders' and users' access to knowledge and information regarding using the program, i.e., training on the mechanics of the program to [Access to Knowledge & Information](http://cfirwiki.net/wiki/index.php?title=Access_to_Knowledge_%26_Information).  Code statements related to engagement strategies and outcomes, e.g., how key stakeholders became engaged with the innovation and what their role is in implementation to [Engaging](http://cfirwiki.net/wiki/index.php?title=Engaging): Key Stakeholders.  Code descriptions of outside group memberships and networking done outside the organization to [Cosmopolitanism](http://cfirwiki.net/wiki/index.php?title=Cosmopolitanism). | 1. Lack of formal communication­_B 2. Alert fatigue_B 3. Staff meetings/engagement_F 4. Alerts/Flagging/Reports_F 5. Top-down information flow_F 6. Alerts/Flagging/Reports_R | 1B. Lack of/poor communication exists between staff; communication occurs as a “through the grapevine” information flow  2B. Includes any negative comments about planned PT alerts expected to not be used or ignored due to high volume of other pop-ups in the system  1F. Staff meetings or engagement strategies exist already in regards to learning new information about new projects/things that are going on in the pharmacy; this is perceived as something which would support future PT implementation.  2F. Specific alerts can be created by PT, reports can be created/printed out by PT – these facilitate implementation of PT.  3F. Information is given to staff from higher-ups in the organization; therefore, it is expected that decision-makers would clearly communicate plans about PT to staff. Includes non-verbal communication as well.  1R. Stakeholder recommendations for specific alerts to be created in the EHR by PatientToc, reports to be created/printed out get coded here. Also code recommendations that all information created in these reports be spread to whatever necessary staff in a timely manner |
| 3C_Culture | Culture_B  Culture_F  Culture_R | Definition: Norms, values, and basic assumptions of a given organization. | 1. Empl_challenge_B 2. Tech_challenge_B 3. Pat_challenges_B 4. Transparency_F 5. Goals_F 6. Org_F | 1B. Code statements which indicate that the ways in which staff roles and competence level influences how work gets done is expected to serve as a barrier to PT. This includes attitude of resistance with corporate or top-down initiated mandates and fear associated with change that includes aversion to risk taking endeavors.  2B. Ways in which technology poses challenges to users which are expected to be barriers to PT, such as savviness of technicians, pharmacists, and patients.  3B. Descriptions of the pharmacy’s patient population such as comments about literacy levels and age-related issues that the stakeholder perceives as a barrier to future PT implementation.  1F. Decisions and programs are made known throughout the organization to a large extent, which is expected to help with PT implementation.  2F. The pharmacy organization and employees set defined quantifiable goals and are motivated by them; this is expected to help PT implementation. Belief that goals and values of patient-centeredness and patient focused services/initiatives and services will facilitate implementation.  3F. Comments that describe positive ways in which the level and extent of cooperation between and among individuals, open-mindedness, and teamwork in organization is expected to support PT implementation. Impact of organization size and layers of management on staff attitude and beliefs, such as more accepting in organizations with fewer layers. |
| 3D_ImplementClimate | ImplementClimate_B  ImplementClimate_F  ImplementClimate_R | Definition: The organization’s capacity for change, shared receptivity of involved individuals to PatientToc, and the extent to which use of PatientToc is or would be rewarded, supported, and expected within their organization.  Inclusion Criteria: Include statements regarding the general level of receptivity to implementing PatientToc.  Code statements regarding the general level of receptivity that are captured in the sub-codes. | 1. Extra work/responsibility_B 2. Staff receptivity_B 3. Neg_work_environment_B 4. Top down pressure_F 5. Staff receptivity_F | 1B. Expected barriers to receptivity because of having to do an extra task (PatientToc)  2B. General negative staff receptivity to implementing PatientToc due to poor attitudes, general resistance to change, dislike of the general idea, etc.  3B. General negative comments regarding tension between staff/ negativity in the work environment. These would be barriers to implementation.  1F. Staff is expected to use PatientToc, whether they want to or not; while this might create feelings of discontent, it is coded as a facilitator because it “forces” compliance with the implementation plan.  2F. General positive staff receptivity to implementing PatientToc due to just liking the idea, general good attitudes, etc. |
| 3D1_TensionforChange | TensionforChange_B  TensionforChange_F  TensionforChange_R | Definition: The degree to which stakeholders perceive the current situation as intolerable or needing change.  Inclusion Criteria: Include statements that do or do not demonstrate a strong need for the current situation to change. Code statements that reveal an untenable situation.  Code statements regarding specific needs of individuals that demonstrate a need for PatientToc, but do not necessarily represent a strong need or an untenable status quo to [Needs and Resources of Those Served by the Organization.](http://cfirwiki.net/wiki/index.php?title=Patient_Needs_%26_Resources)  Code statements that demonstrate PatientToc is better (or worse) than existing programs to [Relative Advantage](http://cfirwiki.net/wiki/index.php?title=Relative_Advantage). | 1. Lack of need/tension_B 2. Desire for change_F | 1B. Stakeholders’ perceptions that there is no need for implementation of PatientToc/no need to change current processes  1F. Stakeholders’ comments demonstrating a desire for change/Want to gather new types of information using PatientToc |
| 3D2_Compatability | Compatability_B  Compatability_F  Compatability_R | Definition: The degree of alignment between values belonging to PatientToc and those belonging to involved individuals (patients, providers/staff). Also includes how PatientToc fits in with existing workflows and systems.  Inclusion Criteria: Include statements that demonstrate the level of compatibility PatientToc has with organizational values and workflow. Include statements that PatientToc did or did not need to be adapted as evidence of compatibility or lack of compatibility.  Code statements regarding the priority of PatientToc based on compatibility with organizational values to [Relative Priority](http://cfirwiki.net/wiki/index.php?title=Relative_Priority), e.g., if an innovation is not prioritized because it is not compatible with organizational values. | 1. Alert_fatig_B 2. Slow_flow_B 3. Integr_B 4. Pt_pop_B 5. Help_flow_F 6. Less paper_F 7. Capt_aud_F 8. MTM_F 9. Paper_alt_R 10. Capt_aud_R 11. MTM_R 12. Demo_R 13. Integr_R 14. Interv_R | 1B. Stakeholders perceive that PatientToc may contribute to the problem of alert fatigue; this belief is a barrier to PT implementation.  2B. Stakeholders are concerned that PatientToc could slow down workflow, especially during busy times. This may be because PatientToc survey is too long and/or the PatientToc and pharmacist will ask the same questions of the patient. This concern is a barrier to implementation.  3B. PatientToc is not integrated with other software in the pharmacy; this is expected to pose a barrier to implementation.  4B. Patient population is not interested in using new technology, not necessarily age-related issues or literacy.  1F. PatientToc is expected to help facilitate aspects of workflow and patient care. This expectation is a facilitator to implementation.  2F. Utilizing PatientToc is expected to decrease paperwork. This expectation is a facilitator to implementation.  3F. PatientToc is expected to fit into workflow if done while patients are waiting for meds, as they are a captive audience. This expectation is a facilitator to implementation.  4F. It is expected that PatientToc could be integrated into MTM sessions with patients. This expectation is a facilitator to implementation.  1R. PatientToc should be considered as an alternative (to paper forms) mechanism for capturing basic “intake” information from patients.  2R. PatientToc PROs should be completed while patients are waiting for meds after they have dropped off their prescriptions, as they are a captive audience.  3R. Use of PatientToc as part of MTM delivery should be considered. Patients could fill out the PatientToc PROs during or prior to their scheduled CMR/MTM encounter.  4R. PatientToc should be considered as a potential tool to update patient demographic information and insurance.  5R. PatientToc should be able to integrate with the software that the pharmacy uses for prescription fulfillment and/or for patient records.  6R. The research team needs to consider where in workflow an intervention would occur and how the pharmacist would be alerted that an intervention is needed. Specific workflow/alert recommendation statements are coded here. |
| 3D3_RelPriority | RelPriority_B  RelPriority_F  RelPriority_R | Definition: Individuals’ shared perception of the importance of PatientToc implementation within the organization.  Inclusion Criteria: Include statements that reflect the relative priority of PatientToc, e.g., statements related to change fatigue in the organization due to implementation of many other programs.  Code statements regarding the priority of PatientToc based on compatibility with organizational values to [Compatibility](http://cfirwiki.net/wiki/index.php?title=Compatibility), e.g., if PatientToc is not prioritized because it is not compatible with organizational values. | 1. Other_proj_B 2. Lack_evid_B 3. Budget_B 4. Need_softw_F 5. Reimburs_F 6. Adhere_F 7. Pt_benefit_R | 1B. Other projects are viewed as more urgent for the pharmacy than PatientToc; this poses a barrier to implementation.  2B. PatientToc is not viewed as a priority without evidence of success to show to management; this poses a barrier to implementation.  3B. Pharmacy doesn’t have the budget to make PatientToc a priority; this poses a barrier to implementation.  1F. Staff indicate that a high priority would be placed on PT implementation due to a perceived need for PatientToc  2F. Staff believe that PatientToc could help improve medication reimbursements; this perception is a facilitator to implementation.  3F. Staff believe that PatientToc could help improve medication adherence; this perception is a facilitator to implementation.  1R. A clear benefit to patients that can be easily communicated to all stakeholders must be demonstrated for subsequent implementation success. The research team should ensure the project is designed to determine this. |
| 3D4_OrgIncentRewards | OrgIncentRewards_B  OrgIncentRewards_F  OrgIncentRewards_R | Definition: Extrinsic incentives such as goal-sharing, awards, performance reviews, promotions, and raises in salary, and less tangible incentives such as increased stature or respect.  Inclusion Criteria: Include statements related to whether organizational incentive systems are in place to foster (or hinder) implementation, e.g., rewards or disincentives for staff engaging in the PatientToc. | 1. No Incentives_B 2. Job Security_F 3. Financial bonuses_F 4. Food bonuses_F 5. Soft Equity_F 6. Financial bonuses_R 7. Food bonuses_R 8. Soft Equity_R 9. Individualized rewards_R 10. Team rewards_R | 1B. No incentives are offered to go “above and beyond” at work or to meet standards given by the pharmacy – people are just expected to “get the job done.” This approach is expected to be a barrier to implementation.  1F. Only site 01/02 – external person had a full time job at the site, needed implementation to keep his job (he tried extra hard to keep implementation going)  2F. Money/gift cards were given (or are expected to be given) to people who implemented PatientToc in a desirable fashion; this was/would be a facilitator to implementation.  3F. Food/lunches were given (or are expected to be given) to people who implemented PatientToc in a desirable fashion; this was/would be a facilitator to implementation.  4F. “soft equity” – Staff reported having (or expecting to have) a fun/good time implementing PatientToc – also includes general comments about how staff enjoys having friendly competitions while meeting metrics and/or general comments about how working together makes team members happier. This enjoyment is an implementation facilitator.  1R. Recommendations for financial rewards for use/implementation of PatientToc  2R. Recommendation for food bonuses for use/implementation of PatientToc  3R. Recommendation to not have rewards, but to improve work environment/ rapport between staff by having a friendly competition  4R. Recommendation for awards to be given to an individual for sustained use of PatientToc (winner of a competition, for example)  5R. Recommendation of awards given to all of the staff for sustained use of PatientToc – example: All staff met a metric to get X amount of PatientTocs done per day and all get an incentive in return |
| 3D5_GoalsFeedback | GoalsFeedback_B  GoalsFeedback_F  GoalsFeedback_R | Definition: The degree to which goals for implementation of PatientToc (not what was loaded into PatientToc) are clearly communicated, acted upon, and results fed back to staff, and alignment of that feedback with goals.  Inclusion Criteria: Include statements related to the (lack of) alignment of implementation and intervention goals of PatientToc as well as feedback to staff regarding those goals, e.g., regular audit and feedback showing any gaps between the current organizational status and the goal. Goals and Feedback can include organizational processes and supporting structures independent of the implementation process that are impacted by PatientToc implementation (e.g., comments about how an existing goal aligns with goals they have for PatientToc implementation.)  Code statements that refer to the implementation team’s assessment of the progress toward and impact of implementation, the outcomes that would need to be measured as part of the evaluation of PatientToc implementation, as well as the interpretation of outcomes related to implementation to [Reflecting & Evaluating](http://cfirwiki.net/wiki/index.php?title=Reflecting_%26_Evaluating). Reflecting and Evaluating is part of the implementation process; it likely ends when implementation activities end.  Similar information could be coded in as Goals/Feedback and Reflecting & Evaluating based on the context. For example, a goal might be that the pharmacy wants to improve Star Ratings and they believe PatientToc aligns with this goal. That statement would be coded as Goals/Feedback. However, they might also recommend formal measurement of Stars Ratings metrics and frequency at which measurement should occur as part of the evaluation of PatientToc implementation and that would be coded as Reflecting & Evaluating. Another example is that they set a goal of having X patients per week complete assessments in PatientToc. This would be coded as Goals/Feedback but if they want to measure the number of patients completing assessments as a formal outcome, code as Reflecting & Evaluating. | 1. Goal and outcome disconnect_B 2. Time need_B 3. Lack of goals_B 4. Goals, progress, accountability_F 5. Align with pharmacy/clinic goals_F 6. Communication_F 7. Goals_R 8. Pharm_goal_align_R 9. Communication_R | 1B. Disconnect felt between actual/expected PT-related goals i.e., goals of number of PatientTocs done versus actual patient care outcomes. This feeling of disconnect is expected to be a barrier to implementation.  2B. Time needed to communicate purpose and benevolence of program to patients. Also includes time needed to educate on proper completion of surveys so counts toward goals.  3B. Staff perceive there is a lack of clearly defined goals (or awareness of such) and this is expected to be a barrier to implementation.  1F. At LA Net sites, having clearly communicated goals for survey completion encourages staff engagement and is a facilitator to PT. Mechanisms such as google doc tracker, board on wall, targeting low numbers tracks progress of use of program. Accountability tracking and assisting as needed to meet goals. Include codes that relate to how this could work in pharmacies.  2F. Stakeholders beliefs and comments about alignment of goals of use of PatientToc with clinic/pharmacy goals such as number of fills and patient satisfaction, including adaptations to survey to continue this process and how this alignment would support/has supported PT implementation.  3F. Communication mechanisms of progress toward goals such as regular meetings, individually has occurred or is expected to occur and supports/would support PT implementation.  1R. Set finite goals for implementation, such as number of patients to complete PT PROs per day, etc.  2R. Align PatientToc implementation goals with existing pharmacy goals such as adherence programs, fill numbers, and patient experience, including ways in which patient feedback is elicited to determine benefit to them and satisfaction and ways in which outcomes to patients and to pharmacist interactions and interventions is impacted by PatientToc.  3R. Ensure that PT-related goals and feedback about goals is communicated clearly to stakeholders. |
| 3D6_LearnClimate | LearnClimate_B  LearnClimate_F  LearnClimate_R | Definition: A climate in which: 1. Leaders express their own fallibility and need for team members’ assistance and input; 2. Team members feel that they are essential, valued, and knowledgeable partners in the change process; 3. Individuals feel psychologically safe to try new methods; and 4. There is sufficient time and space for reflective thinking and evaluation.  Inclusion Criteria: Include statements that support (or refute) the degree to which key components of an organization exhibit a “learning climate.” | 1. Open_change_F 2. CE_F 3. Students_F | 1F. Pharmacy is generally open to trying new ideas which would facilitate PT implementation  2F. Opportunities for CE are provided in this environment which demonstrates a commitment by the pharmacy to learn new things which is believed to facilitate PT implementation.  3F. Pharmacy is willing to take and teach students which demonstrates the pharmacy values education/innovation which is believed to facilitate PT implementation. |
| 3E_ReadinessImplement | ReadinessImplement_B  ReadinessImplement_F  ReadinessImplement_R | Definition: Tangible and immediate indicators of organizational commitment to its decision to implement PatientToc.  Inclusion Criteria: Include statements regarding the general level of readiness for implementation of PatientToc.  Code statements regarding the general level of readiness for implementation that are captured in the sub-codes. | 1. General_optimism_F | 1F. General optimism of staff will help facilitate the implementation of PatientToc. |
| 3E1_LeadershipEngage | LeadershipEngage_B  LeadershipEngage_F  LeadershipEngage_R | Definition: Commitment, involvement, and accountability of leaders and managers with the implementation of PatientToc (Includes pharmacy managers, owners, CMOs (LA Net), formal leaders within organization).  Inclusion Criteria: Include statements regarding the level of engagement of organizational leadership. E.g. How much leaders are “buying in” to PatientToc.  ( Include statements about getting leaders to “buy in” to PatientToc.)  Code statements about leadership’s opinion influencing others in the Organization to Opinion Leaders.  Code statements regarding leadership engagement to Engaging: [Formally Appointed Internal Implementation Leaders](http://cfirwiki.net/wiki/index.php?title=Formally_Appointed_Internal_Implementation_Leaders) or [Champions](http://cfirwiki.net/wiki/index.php?title=Champions) *if* an organizational leader is also an implementation leader, e.g., if a director of primary care takes the lead in implementing a new treatment guideline. Note that a key characteristic of this Implementation Leader/Champion is that s/he is also an Organizational Leader. | 1. Leadership_disconnect_B 2. Leadership_values_F 3. Manage_style_F 4. Key_stakeholders_F 5. Accountability_R 6. Benefit_patient_R 7. Value_staff_R | 1B. One leader has buy-in while another is skeptical; this is expected to be a barrier to PT implementation.  1F. How those in charge show values and what those values are such as pushing for excellence, being supportive, and present and at the table. These demonstrated values are expected to facilitate PT implementation.  2F. Examples of positive ways people are managed such as not micro-managing and instead trusting the staff, how accountability is handled, and demonstrating that team-based approaches are valued. These behaviors are expected to facilitate PT implementation.  3F. Having key people such as lead tech currently involved in other initiatives is expected to facilitate PT implementation.  1R. Encourage decision-makers to hold pharmacy staff accountable by providing motivation and feedback to pharmacy staff.  2R. Demonstrate that the program is going to benefit patients in order to get buy in from leadership.  3R. Ensure the study/implementation plan is designed so that it is most likely to demonstrate that PT implementation is going to benefit staff and workflow. |
| 3E2_AvailResources | AvailResources_B  AvailResources_F  AvailResources_R | Definition: The level of resources an organization dedicated for implementation of PatientToc and on-going operations including physical space and time.  Inclusion Criteria: Include statements related to the presence or absence of resources specific to PatientToc implementation.  Code statements related to training and education t.  Code statements related to the quality of materials to [Design Quality & Packaging](http://cfirwiki.net/wiki/index.php?title=Design_Quality_%26_Packaging).  In a research study, exclude statements related to resources needed for conducting the research components (e.g., time to complete research tasks, such as IRB applications, consenting patients). | 1. Staff time_B 2. Tech support_B 3. Staff Time_F 4. Tech Support_F 5. Staff Time_R 6. Tech Support_R | 1B. Lack of time/ lack of people available to implement PatientToc. This would be a barrier to implementation. Includes statements related to not having enough staff available.  2B. Lack of tech support for implementing PatientToc – also general negative comments about tech support that may have been available for other projects at the site  1F. Staff/Time that would be needed is available for implementing PatientToc  2F. Sufficient amount of tech support was provided/is expected to be given for implementation of PatientToc – also includes general positive comments about tech support that may have been done in other projects at the site  1R. Recommendation that extra staff/time be provided/available to implement PatientToc  2R. Recommendation that outside tech/IT support for PatientToc from the PatientToc company itself be available to support PT implementation. |
| 3E3_AccessKnowlInfo | AccessKnowlInfo_B  AccessKnowlInfo_F  AccessKnowlInfo_R | Definition: Ease of access to digestible information and knowledge about PatientToc and how to incorporate it into work flow.  Inclusion Criteria: Include statements related to implementation leaders' and users' access to knowledge and information regarding use of PatientToc, i.e., training on the mechanics of the program.  Code statements related to engagement strategies and outcomes, e.g., how key stakeholders became engaged with PatientToc and what their role is in implementation to [Engaging](http://cfirwiki.net/wiki/index.php?title=Engaging): Key Stakeholders.  Code statements about general networking, communication, and relationships in the organization, such as descriptions of meetings, email groups, or other methods of keeping people connected and informed, and statements related to team formation, quality, and functioning to [Networks & Communications](http://cfirwiki.net/wiki/index.php?title=Networks_%26_Communications). | 1. Train_B 2. Train_F   1. Train_R   1. CE_R 2. IT_R | 1B. Staff had/currently have a lack of training or familiarity with PatientToc so this would be a barrier to implementation .  1F. Sufficient training was/is expected to be provided for PatientToc and found/expected to be helpful in supporting implementation.  1R. Web based training and hands on training should be used when possible as these are believed to be best for staff. Include recommendations for other types of training or general sentiment that training would be recommended for implementation.  2R. Training for PatientToc should be worth CE credits and may be provided through lunch and learns.  3R. IT support should be made available to make training possible |
| **4. Characteristics of Individuals** |  |  |  |  |
| 4A_KnowBeliefsInterven | KnowBeliefsInterven_B  KnowBeliefsInterven_F  KnowBeliefsInterven_R | Definition: Individuals’ attitudes toward and value placed on PatientToc, as well as familiarity with facts, truths, and principles related to PatientToc.  Inclusion Criteria:  Code statements related to familiarity with evidence about PatientToc to [Evidence Strength & Quality](http://cfirwiki.net/wiki/index.php?title=Evidence_Strength_%26_Quality) | N/A | N/A |
| 4B_SelfEfficacy | SelfEfficacy_B  SelfEfficacy_F  SelfEfficacy_R | Definition: Individual belief in their own capabilities to execute courses of action to achieve implementation goals.  Inclusion Criteria: | 1. Confidence_F | 1F. Positive comments about the ease of use/confidence of staff and patients to use PatientToc without help from someone else as this high self-efficacy is viewed as a facilitator to implementation |
| 4C_IndividStageChange | IndividStageChange_B  IndividStageChange_F  IndividStageChange_R | Definition: Characterization of the phase an individual is in, as s/he progresses toward skilled, cooperative, and sustained use of PatientToc.  Inclusion Criteria: | 1. Aversion_B 2. Receptive_F | 1B. Staff and patients have an aversion to change which would be a barrier to PT implementation.  1F. Staff and patients are receptive to change which is a facilitator to PT implementation. |
| 4D_IndividIDwithOrg | IndividIDwithOrg_B  IndividIDwithOrg_F  IndividIDwithOrg_R | Definition: A broad construct related to how individuals perceive the organization, and their relationship and degree of commitment with that organization.  Inclusion Criteria: | 1. Staff_stabil_B 2. New_client_B 3. Part of community_F 4. Loyalty to pharmacy_F 5. Shared decisions_F | 1B. Things that create less stable work environment such as staff turnover and looking for other jobs. These changes in the work environment are expected to serve as barriers to PT implementation.  2B. Many new patients so many not have complete prescription profiles or time to build relationships and this is expected to be a barrier to PT implementation.  1F. Ways in which the organization shows commitment to the community it serves; evidence of commitment to the community is expected to support PT implementation.  2F. Shows staff or patient is dedicated to the pharmacy even with adversity and change and this loyalty is expected to support PT implementation.  3F. Value in being part of decision process that leads to positive relationship with organization; this is expected to support PT implementation. |
| 4E_OtherPersonalAttr | OtherPersonalAttr_B  OtherPersonalAttr_F  OtherPersonalAttr_R | Definition: A broad construct to include other personal traits such as tolerance of ambiguity, intellectual ability, motivation, values, competence, capacity, and learning style.  Inclusion Criteria: | 1. Passivity_B 2. Motivation_F | 1B. Passivity of staff is indicated and expected to be a barrier to PT implementation.  1F. Motivation of staff appears to be strong and is expected to be a facilitator to PT implementation. |
| **5. Process** |  |  |  |  |
| 5A_Planning | Planning_B  Planning_F  Planning_R | Definition: The degree to which and quality of a scheme or method of behavior and tasks for implementing PatientToc are developed in advance.  Inclusion Criteria: Include evidence of pre-implementation diagnostic assessments (for LA Net sites only; the current research Aim 1 IS the diagnostic assessment for the pharmacy use of PatientToc) and planning, as well as refinements to the plan.  AND include General comments about planning for PatientToc implementation in community pharmacies. | 1. Time_constr_B 2. Workflow_R 3. Pt_edu_R | 1B. PatientToc may be difficult to use due to time constraints in a pharmacy on the part of staff and/or patients.  1R. Recommendations as to when in the workflow would be ideal for PatientToc to be used.  2R. Recommendation to provide patient education on the PatientToc tablets |
| 5B_ Engaging | Engaging_B  Engaging_F  Engaging_R | Definition: Attracting and involving appropriate individuals in the implementation and use of PatientToc through a combined strategy of social marketing, education, role modeling, training, and other similar activities.  Inclusion Criteria: Include statements related to engagement strategies and outcomes. Also code quality of engagement.  Code statements related to specific sub constructs, e.g., [Champions](http://cfirwiki.net/wiki/index.php?title=Champions) or [Opinion Leaders](http://cfirwiki.net/wiki/index.php?title=Opinion_Leaders) as such.  Code statements related to who participated in the decision process to implement PatientToc to [Innovation Source](http://cfirwiki.net/wiki/index.php?title=Intervention_Source), as an indicator of internal or external innovation source. | 1. Pat_challenges_B   1. Phased_roll-out_F  2. Awareness_F   1. Awareness_R 2. Key_stakeholders_R | 1B. Challenges faced by patients with engagement, or perceived to be a barrier to engagement with PT. Ability of patients to understand and correctly engage with tablet in the first place is perceived as a likely barrier to PT implementation. Perceived inability to be in contact with patients in a way that facilitates involvement in PT i.e., patients that get delivery or have caregivers. Concerns that patients won’t see the value in PT and completing the PROs, including care expectations at pharmacy, wanting to engage with people vs technology, etc.  1F. The belief that phased roll-out such as beginning use of PT with one patient-group before adding another such as those with high needs, regular customers, or those mandated to fill out particular forms anyway has facilitated engagement with PT.  2F. The belief that ways in which program is introduced and explained to patient including use of champion or someone patient trusts and will listen to, advertising, telling them about it ahead of time, and use of other departments. facilitates implementation of PT.1  1R. Recommendations for ways to best introduce and explain PT to patients, including use of champions or someone patients trust and will listen to. Importance of addressing issues quickly so outcomes can be realized and seen. Code recommendations for ways PT will be made known to patients such as advertising, telling them about it ahead of time, and use of other departments.  2R. Ways in which other key individuals can be utilized such as staff, delivery personnel, and other patients. |
| 5B1_OpinionLeaders | OpinionLeaders_B  OpinionLeaders_F  OpinionLeaders_R | Definition: Individuals in an organization that have formal or informal influence on the attitudes and beliefs of their colleagues with respect to implementing PatientToc.  Inclusion Criteria: Include statements related to engagement strategies and outcomes, e.g., how the opinion leader became engaged (e.g., in the case of LA Net clinics) OR would become engaged with PatientToc and what their role is/was/would be in implementation. | N/A | N/A |
| 5B2_FormalImplementLeaders | FormalImplementLeaders_B  FormalImplementLeaders_F  FormalImplementLeaders_R | Definition: Individuals from within the organization who have been/or would be expected to formally appointed with responsibility for implementing PatientToc as coordinator, project manager, team leader, or other similar role.  Inclusion Criteria: Include statements related to engagement strategies and outcomes, e.g., how the formally appointed internal implementation leader became engaged with the PatientToc and what their role is in implementation.  Code statements regarding leadership engagement to [Leadership Engagement](http://cfirwiki.net/wiki/index.php?title=Leadership_Engagement) *if* an implementation leader is also an organizational leader, e.g., if a director of primary care takes the lead in implementing a new treatment guideline. | 1. CMO_F 2. Manag_direct_F 3. Lead tech_F 4. Pharm_R 5. Manag_direct_R 6. Tech_R | 1F. The CMO (chief of medical staff) is a formal implementation leader and this positively contributed to successful PT implementation at LA Net sites.  2F. The pharmacy manager or pharmacy director or Pharmacist in charge (PIC) is expected to be formal implementation leader for PT or has been a formal implementation leader for prior programs and this is expected to support successful PT implementation.  3F. The lead pharmacy technician is expected to be formal implementation leader for PT or has been a formal implementation leader for prior programs and this is expected to support successful PT implementation.  1R. Recommend appointing pharmacist(s) as formal implementation leader(s).  2R. Recommend appointing the pharmacy manager/director as a formal implementation leader.  3R. Recommend appointing a pharmacy technician(s) as formal implementation leader(s). |
| 5B3_Champions | Champions_B  Champions_F  Champions_R | Definition: “Individuals who dedicate themselves to supporting, marketing, and ‘driving through’ [PatientToc]”, overcoming indifference or resistance that PatientToc may provoke in an organization.  Inclusion Criteria: Include statements related to engagement strategies and outcomes, e.g., how the “champion” became engaged with the PatientToc and what their role is in implementation.  Code statements regarding leadership engagement to [Leadership Engagement](http://cfirwiki.net/wiki/index.php?title=Leadership_Engagement) *if* a champion is also an organizational leader, e.g., if a director of primary care takes the lead in implementing a new treatment guideline. | 1. Team-based_B 2. No need_B 3. Formal leader_F 4. Process _F 5. Non-traditional_F 6. Process_R | 1B. One person would not champion so this is expected to create a barrier to PT implementation.  2B. Stakeholders do not perceive a need for champion and this is expected to create a barrier to PT implementation.  1F. A role of leadership such as pharmacist, tech supervisor, or medical director has been/expected to be involved in championing for PT which is viewed as a facilitator to implementation.  2F. Delineates steps that person does to champion, includes if asking for volunteers, voluntold, informal vs formal. One person that stays on top of program, formal or informal. Having a clear process already in mind is viewed as a facilitator to PT implementation.  3F. Roles outside of leadership but internal to organization including quality improvement or cashier that stakeholders comment on playing/expected to play a critical role in supporting PT implementation. Roles such as students, residents, or someone outside of traditional pharmacist or staff role that interacts with patients expected to play a role in supporting PT implementation.  1R. Recommendation to ensure a clear process for how PT will get championed such as leading several others, pragmatic considerations, emergence of natural leader. |
| 5B4_ExtChgAgents | ExtChgAgents_B  ExtChgAgents_F  ExtChgAgents_R | Definition: Individuals who are affiliated with an outside entity who formally influence or facilitate PatientToc decisions in a desirable direction.  Inclusion Criteria: Include statements related to engagement strategies and outcomes about individuals who are external to the company and influence/facilitate decisions related to PatientToc e.g., how the external change agent (entities outside the organization that facilitate change) became engaged with the PatientToc and what their role is in implementation, e.g., how they supported implementation efforts.  Note: It is important to clearly define what roles are external and internal to the organization. Code statements regarding facilitating activities, such as training in the mechanics of the program to [Access to Knowledge & Information](http://cfirwiki.net/wiki/index.php?title=Access_to_Knowledge_%26_Information) *if* the change agent is considered internal to the study, e.g., a staff member at the national office. If the study considers this staff member internal to the organization, it should be coded to [Access to Knowledge & Information](http://cfirwiki.net/wiki/index.php?title=Access_to_Knowledge_%26_Information), even though their support may overlap with what would be expected from an External Change Agent. | 1. IT help_B 2. Specialized_F 3. IT help_F 4. Specialized_R 5. IT help_R 6. QI_R | 1B. IT help already available at the pharmacy is expected to be insufficient for successful PT implementation.  1F. Specialized PatientToc help/representative available, specifically with one LA Net site.  2F. Extra IT help is available and/or perceived as helpful for PT implementation.  1R. Recommendation to have a specialized PatientToc representative available, similar to the one LA Net site.Also includes recommendations to NOT have a specialized PatientToc representative  2R. Recommendation to having extra IT support available.  3R. Recommendation to have a QI person and/or someone to run data reports available. |
| 5B5_KeyStakeholders | KeyStakeholders_B  KeyStakeholders_F  KeyStakeholders_R | Definition: Individuals from within the organization that are directly impacted by PatientToc, e.g., pharmacy staff responsible for getting patients to use PatientToc for PROs.  Inclusion Criteria: Include statements related to engagement strategies and outcomes about/by providers/staff. e.g., how providers/staff became engaged with PatientToc and what their role is in implementation.  Code statements related to implementation leaders' and users' access to knowledge and information regarding using the program, i.e., training on the mechanics of the program to [Access to Knowledge & Information](http://cfirwiki.net/wiki/index.php?title=Access_to_Knowledge_%26_Information).  Code statements about general networking, communication, and relationships in the organization, such as descriptions of meetings, email groups, or other methods of keeping people connected and informed, and statements related to team formation, quality, and functioning to [Networks & Communications](http://cfirwiki.net/wiki/index.php?title=Networks_%26_Communications) | 1. Technicians_F 2. Pharmacists_F 3. Technicians_R 4. Pharmacists_R | 1F. Technicians would be expected (based on prior experiences with new initiatives) to help with implementation of PatientToc – this would be a facilitator to implementation  2F. Pharmacists would be expected (based on prior experiences with new initiatives) to help with implementation of PatientToc – this would be a facilitator to implementation  1R. Recommendation for technicians to be the ones to mainly implement/show PatientToc to patients  2R. Recommendation for pharmacists to be the ones to implement/show PatientToc to patients |
| 5B6_InterventionParticipants | InterventionParticipants_B  InterventionParticipants_F  InterventionParticipants_R | Definition: Individuals served by the organization (i.e., patients) that are the end-users (intended audience) of PatientToc.  Inclusion Criteria: Include statements related to engagement strategies and outcomes about/by patients, e.g., how PatientToc participants became engaged with PatientToc.  Code statements demonstrating (lack of) awareness of the needs and resources of those served by the organization and whether or not that awareness influenced the implementation or adaptation of PatientToc to [Needs & Resources of Those Served by the Organization](http://cfirwiki.net/wiki/index.php?title=Patient_Needs_%26_Resources). | 1. Data theft_B 2. Anonymity_B 3. Tech_engage_B 4. Comm_engage_F 5. Randomize_R 6. PR_R 7. Incentiv_R 8. Explain_R | 1B. Patients are concerned about data security and theft in regards to PatientToc. This concern would likely be a barrier to PT implementation.  2B. Patients are concerned about their responses being traced back to them, they would prefer anonymity. This concern would likely be a barrier to PT implementation.  3B. A number of patients do not want to talk about their meds, just a quick trip to the pharmacy. Patients may also prefer human engagement over using tablet technology. These preferences would likely be a barrier to PT implementation.  1F. Participating in PatientToc is viewed by patients as a form of community engagement; this viewpoint is a facilitator to PT implementation. Also code here general willingness to participate in surveys and feedback with pharmacy.  1R. Randomize patient participation in the PatientToc tablet. Patients thought this might help put others at ease and not make it appear as if certain patients were being “targeted”.  2R. Recommendation to use PR and marketing to help explain the purpose of PatientToc and therefore increase patient engagement.  3R. Recommendation to incentivize patient participation with PatientToc tablet.  4R. Providing an explanation to patients as to why PatientToc is being implemented will help with engagement. |
| 5C_Executing (LA Net sites only) | Executing_B  Executing_F  Executing_R | Definition: Carrying out or accomplishing the implementation of PatientToc according to plan.  Inclusion Criteria: Include statements that demonstrate how implementation occurred with respect to the implementation plan. | N/A | N/A |
| 5D_ReflectandEvaluate | ReflectandEvaluate_B  ReflectandEvaluate_F  ReflectandEvaluate_R | Definition: Quantitative and qualitative feedback (i.e., team debriefs, implementation outcomes, about the progress and quality of the implementation of PatientToc accompanied with regular personal and team debriefing about progress and experience. Includes statements of how stakeholders would want to measure the implementation of PatientToc.  Inclusion Criteria: Include statements that refer to the implementation team’s (lack of) assessment of the progress toward and impact of implementation, as well as the interpretation of outcomes related to implementation. Reflecting and Evaluating is part of the implementation process; it likely ends when implementation activities end.  Similar information could be coded in as Goals/Feedback and Reflecting & Evaluating based on the context. For example, a goal might be that the pharmacy wants to improve Star Ratings and they believe PatientToc aligns with this goal. That statement would be coded as Goals/Feedback. However, they might also recommend formal measurement of Stars Ratings metrics and frequency at which measurement should occur as part of the evaluation of PatientToc implementation and that would be coded as Reflecting & Evaluating. Another example is that they set a goal of having X patients per week complete assessments in PatientToc. This would be coded as Goals/Feedback but if they want to measure the number of patients completing assessments as a formal outcome, code as Reflecting & Evaluating.  Code statements related to the (lack of) alignment of implementation and PatitnetToc goals with larger organizational goals, as well as feedback to staff regarding those goals, e.g., regular audit and feedback showing any gaps between the current organizational status and the goal to [Goals & Feedback](http://cfirwiki.net/wiki/index.php?title=Goals_%26_Feedback). Goals and Feedback may include organizational processes and supporting structures independent of the implementation process but influenced by PatientToc implementation.  Code statements that capture reflecting and evaluating that participants may do during the interview, for example, related to the success of the implementation to [Knowledge & Beliefs about the Innovation](http://cfirwiki.net/wiki/index.php?title=Knowledge_%26_Beliefs_about_the_Intervention). | 1. # interventions made_R 2. Pt satisfaction_R 3. Cost_R 4. Pt outcomes_R 5. Participation rate_R | 1R. The want/need to know how many interventions were made because of PatientToc  2R. The want/need to see how patient satisfaction is affected by implementation of PatientToc  3R. Want/need to see how costs/time used was affected by PatientToc implementation. This includes efficiency.  4R. The want/need to see how patient outcomes such as medication adherence is impacted including metrics.  5R. The need/want to see how many patients agree to participate with PT. |
